# Transdermal Delivery of Pramipexole Using Microneedle Technology for the Potential Treatment of Parkinson’s Disease

**DOI:** 10.1021/acs.molpharmaceut.4c00065

**Published:** 2024-04-11

**Authors:** Mary B. McGuckin, Aaron R.J. Hutton, Ellie R. Davis, Akmal H.B. Sabri, Anastasia Ripolin, Achmad Himawan, Yara A. Naser, Rand Ghanma, Brett Greer, Helen O. McCarthy, Alejandro J. Paredes, Eneko Larrañeta, Ryan F. Donnelly

**Affiliations:** † School of Pharmacy, Queen’s University Belfast, Medical Biology Centre, 97 Lisburn Road, Belfast BT9 7BL, United Kingdom; ⊥ Institute for Global Food Security, School of Biological Sciences, Queen’s University Belfast, 19 Chlorine Gardens, Belfast BT9 5DL, United Kingdom; # The International Joint Research Centre on Food Security (IJC-FOODSEC), 113 Thailand Science Park, Pahonyothin Road, Khong Luang ,Pathum Thani12120, Thailand

**Keywords:** pramipexole, dissolving microneedle, hydrogel-forming microneedle, directly compressed tablet, transdermal, Parkinson’s disease

## Abstract

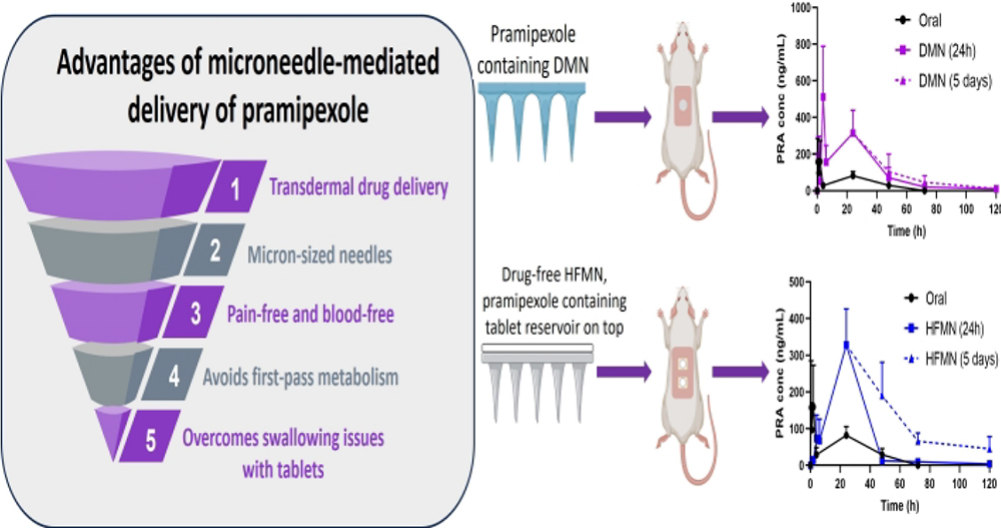

Parkinson’s disease (PD) is a debilitating neurodegenerative disease primarily impacting neurons responsible for dopamine production within the brain. Pramipexole (PRA) is a dopamine agonist that is currently available in tablet form. However, individuals with PD commonly encounter difficulties with swallowing and gastrointestinal motility, making oral formulations less preferable. Microneedle (MN) patches represent innovative transdermal drug delivery devices capable of enhancing skin permeability through the creation of microconduits on the surface of the skin. MNs effectively reduce the barrier function of skin and facilitate the permeation of drugs. The work described here focuses on the development of polymeric MN systems designed to enhance the transdermal delivery of PRA. PRA was formulated into both dissolving MNs (DMNs) and directly compressed tablets (DCTs) to be used in conjunction with hydrogel-forming MNs (HFMNs). In vivo investigations using a Sprague–Dawley rat model examined, for the first time, if it was beneficial to prolong the application of DMNs and HFMNs beyond 24 h. Half of the patches in the MN cohorts were left in place for 24 h, whereas the other half remained in place for 5 days. Throughout the entire 5 day study, PRA plasma levels were monitored for all cohorts. This study confirmed the successful delivery of PRA from DMNs (*C*_max_ = 511.00 ± 277.24 ng/mL, *T*_max_ = 4 h) and HFMNs (*C*_max_ = 328.30 ± 98.04 ng/mL, *T*_max_ = 24 h). Notably, both types of MNs achieved sustained PRA plasma levels over a 5 day period. In contrast, following oral administration, PRA remained detectable in plasma for only 48 h, achieving a *C*_max_ of 159.32 ± 113.43 ng/mL at 2 h. The HFMN that remained in place for 5 days demonstrated the most promising performance among all investigated formulations. Although in the early stages of development, the findings reported here offer a hopeful alternative to orally administered PRA. The sustained plasma profile observed here has the potential to reduce the frequency of PRA administration, potentially enhancing patient compliance and ultimately improving their quality of life. This work provides substantial evidence advocating the development of polymeric MN-mediated drug delivery systems to include sustained plasma levels of hydrophilic pharmaceuticals.

## 1 Introduction

Among neurological disorders, Parkinson’s disease (PD) is the fastest growing in prevalence, disability, and deaths, due to a growing and aging population.^1^ Involuntary movements known as dyskinesias and painful muscle contractions referred to as dystonias often result in speech and mobility issues.^2^ These symptoms contribute significantly to increased disability rates and the requirement for care. Approximately 145,000 people have been diagnosed with PD in the UK, and this figure is estimated to increase by a fifth by 2030.^3^ PD is primarily characterized by the loss of dopaminergic neurons projecting from the substantia nigra located in the brain. Pramipexole (PRA) is a nonergot dopamine agonist, producing its benefit by acting directly on dopamine receptors to imitate a neurotransmitter.^4^ PRA is a white powder, and the base form has a molecular weight of 211.33 Da, making it a small molecule, with its corresponding dihydrochloride monohydrate salt slightly larger at 302.3 Da. It has a high level of absorption and is extensively distributed, indicated by an oral bioavailability of 90% and a volume of distribution of 500 L.^5,6^ At ambient temperature, the parent compound and salt exhibit water solubilities of approximately 12 and 200 mg/mL, respectively.^7^ PRA is currently formulated as a tablet, with immediate release and sustained release variations available on the market containing the salt form of the drug. For Parkinson’s disease treatment, immediate-release PRA tablets are given three times a day up to a maximum of 3.3 mg daily. In contrast, sustained-release tablets are administered once daily with a maximum dose of 3.15 mg per day.^8^ However, there are disadvantages of taking PRA orally as a large proportion of PD patients are required to take medication daily to manage nonmotor symptoms, adding to pill fatigue. Furthermore, PD patients often have delayed gastric emptying leading to decreased stomach motility that eventually affects gut transit. Over time, intestinal absorption of orally administered PRA is slowed, reducing the effectiveness of treatment.^9^ More than 80% of patients with PD develop dysphagia during the course of their disease, rendering oral administration unsuitable.^10^ Exploring an alternative delivery method for PRA, aside from the oral route, could potentially enhance the efficacy of treatment.

The skin is the largest organ in the human body with a surface area encompassing 1.79 m^2^ for an average adult.^11,12^ Skin is an attractive site for drug delivery due to its large surface area, ease of access, and vascularization. There are many advantages of this drug delivery approach as it eliminates pain, discomfort, infection, and the need for trained personnel accompanying other routes such as injections. First-pass hepatic metabolism, which occurs following oral administration, is avoided, consequently improving the bioavailability of drugs. Gastrointestinal degradation and food-related inconsistency in absorption are prevented. Additionally, prolonged release can be achieved, enabling constant plasma concentrations over a period of days or weeks when the same patch remains on the skin.^13^ However, barrier properties of the stratum corneum impose significant restrictions on the successful accomplishment of transdermal drug delivery. As a result, passive drug delivery through the skin is limited to drugs with specific physiochemical properties: molecular weight <500 Da, adequate lipophilicity, and low melting point.^14^

Microneedles (MNs) refer to micron-sized needles ranging from 50 to 900 μm in height that protrude outward from and perpendicular to a flat baseplate.^15^ These needles can penetrate the stratum corneum, and following insertion into the skin, they create microconduits through which drugs can be delivered.^16^ By overcoming the barrier issues associated with delivery through the stratum corneum, MNs provide a promising strategy to enhance transdermal drug delivery. MNs offer many advantages over currently available routes of administration. As an alternative to hypodermic needles, MNs decrease the invasive nature of needles, increasing patient acceptance among individuals with needle phobia. The shaft of MNs has a sufficient length to penetrate the stratum corneum but does not extend deep enough to reach the underlying nerve endings and blood capillaries. As a result, using MNs is virtually devoid of pain and blood. There is a possibility of self-administration of MN patches without the need for trained medical professionals. MNs eliminate sharp waste production, minimizing the risk of transmitting infectious diseases through needle-related accidents. Controlled therapeutic delivery can be achieved, coupled with rapid treatment cessation upon MN patch removal, which increases patient adherence and treatment compliance. Furthermore, MNs bypass first-pass metabolism, resulting in increased drug bioavailability.^17^

This work focuses on the use of polymeric MNs, specifically dissolving MNs (DMNs) and hydrogel-forming MNs (HFMNs). In DMNs, the drug is dispersed within the MN matrix that is composed of dissolvable or degradable biocompatible polymers. Following a simple, one-step application process to the skin, the needles penetrate the stratum corneum and make contact with interstitial fluid beneath the outer layer of the skin. The polymer forming the bulk of the needle begins to dissolve, releasing the entrapped therapeutic within. The choice of polymer allows the rate of release to be controlled. Employing water-soluble polymers facilitates immediate drug release, whereas hydrophobic polymers enable sustained drug delivery as it takes time for the hydrophobic polymer to degrade.^18^ Drug distribution within the needles must be homogeneous, and needles must have sufficient mechanical strength to pierce the stratum corneum, which present a challenge during development.^19^ DMNs have been successfully employed to deliver hydrophobic drugs such as levonorgestrel,^20^ hydrophilic dugs including doxorubicin hydrochloride,^21^ vaccines,^22^ and insulin,^23^ emphasizing their versatility in drug delivery. Furthermore, MNs allow transdermal delivery of water-soluble drugs at higher doses than might be possible with a conventional transdermal patch.^24^ Maximizing drug loading is imperative when developing a DMN patch to ensure that the resulting patch size is as small as possible. Small patch sizes help to facilitate consistent MN insertion into skin, and they are discreet, which are important as patient acceptability and compliance are vital for treating any condition. Considering these factors, formulating DMNs using PRA salt (PRAS) was deemed appropriate due to its higher solubility in water compared to PRAB. When PRAS is formulated with hydrophilic polymers such as PVA and PVP, it was anticipated that they would form a homogeneous blend suitable for casting on to molds. This would facilitate the integration of PRAS into both the needle tips and baseplate, allowing for maximum drug loading. In contrast, more hydrophobic drugs are generally cased into the needle tips and not the baseplate. As such, incorporation of drug in the needle tip and baseplate would result in a higher drug loading and a small patch size.

Swellable MN patches, more commonly known as hydrogel-forming MNs (HFMNs), consist of cross-linked polymers that swell following uptake of interstitial fluid. A separately formulated drug-containing reservoir located on top of the MN patch begins to disintegrate or dissolve once in contact with aqueous fluid and diffuses through the swollen hydrogel matrix into the viable epidermis. This unique approach enables increased drug loading, and the modification of cross-linking within the polymers allows the rate of drug release to be controlled.^25^ Although there are many drug reservoir options, this work describes the formulation of PRA-containing directly compressed tablets (DCTs). DCTs can be used during formulation of high-potency and nonpotent drugs, and the drug content is usually less than 30% of the formulation.^26,27^ In the preparation of tablets, direct compression has many advantages over wet and dry granulation due to fewer processing stages and the elimination of heat and moisture effects, resulting in fewer stability issues and the need for less excipients. Excipients included in DCTs generally include binders and disintegrants.^28^ The use of DCTs in combination with HFMNs was first reported for the delivery of low-molecular-weight drugs including amoxicillin and levofloxacin, where therapeutically relevant concentrations were found in rats following an in vivo study.^27^ Using DCTs extends the spectrum of drug molecules that can be delivered across the skin via MN technology. During the work outlined here, PRA base (PRAB) was chosen to be formulated into DCTs. The more hydrophobic form of PRA (PRAB) was chosen as it was hoped that it would dissolve slowly, with the ultimate aim of sustaining PRA delivery.

The overall aim of this work was to investigate the possibility of transdermal delivery of PRA using DMNs and HFMN devices to potentially improve the treatment of PD. The work described here focuses on formulating the salt form of PRA (PRAS) into DMNs with rapidly dissolving polymers to achieve immediate drug release and the more hydrophobic base form (PRAB) into DCTs to be used with HFMNs with the aim of sustaining PRA delivery. Although DMNs are extremely beneficial and the most suitable MN platform for immediate delivery of low-molecular-weight, water-soluble, and potent molecules such as PRA, it is difficult to sustain the delivery of hydrophilic compounds using DMNs even when slowly degrading polymers such as poly(lactide-*co*-glycolic acid) (PLGA) are employed.^29^ Both DMNs and HFMN patches avoid the need for disposal of sharps waste, resulting in a significant advantage and generating substantial research efforts in the field.

## 2 Materials and Methods

### 2.1 Materials

PRA base (PRAB) and salt (PRAS) were purchased from Cangzhou Enke Pharma-tech Co. Ltd. (China). Acetonitrile (>99.9%), phosphate-buffered saline (PBS) tablets (pH 7.4), PVA 9–10 kDa, PVA 31–50 kDa, PEG10,000, sorbitol, and trifluoracetic acid (TFA) were purchased from Sigma-Aldrich (Dorset, UK). Gantrez S-97 and PVP (MW 58 kDa), sold under the product brand name Plasdone K-29/32, were obtained from Ashland (Worcestershire, UK). Microcrystalline cellulose (MCC) was purchased from DFE Pharma (Klever Strasse, Germany). Anhydrous citric acid and anhydrous sodium carbonate (Na_2_CO_3_) were obtained from BDH Laboratory Supplies (Dorset, UK). LCMS/MS grade methanol and formic acid were purchased from Sigma-Aldrich (Gillingham, UK), with ultrapure water (18.2MΩ-cm) produced in-house using a Millipore water purification system (Millipore, Cork, Ireland).

### 2.2 Dissolving Microneedles Containing Pramipexole Salt (PRAS)

#### 2.2.1 Fabrication of Pramipexole Salt-Containing Dissolving Microneedles

**Figure 1:**
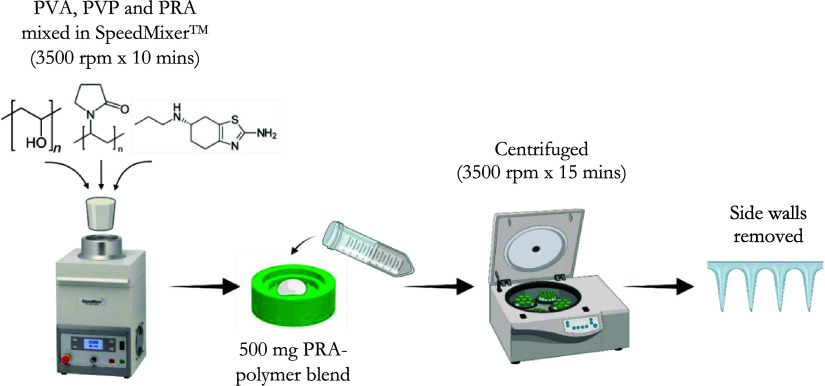
Diagrammatic representation of the casting process to form PRAS-containing DMNs.

Various PRAS-containing DMNs were formulated, with differing drug concentrations and polymer compositions. The most favorable PRAS-containing DMNs were composed of PRAS, 20% w/w PVP 58 kDa, and 15% w/w PVA 9–10 kDa. Using a DAC 150 FVZ SpeedMixer (High Wycombe, England), the PRA-polymer blend was mixed at 3500 rpm for 10 min to obtain a homogeneous mixture. The final aqueous blend contained 10% w/w PRAS and 90% w/w of the polymer mixture. After mixing, 500 mg of the resulting formulation was placed onto silicone micromold templates (LTS Lohmann Therapie-Systeme, Andernach, Germany), leading to the presence of the drug within the needle tips and the baseplate. The micromold templates consisted of 600 pyramidal needles per 0.75 cm^2^, height 750 μm, and base 300 × 300 μm with an interspace of 50 μm. Subsequently, the molds were centrifuged at 3500 rpm for 15 min followed by drying at ambient temperature for 48 h. Finally, the side walls were removed using scissors. A summary of the process can be found in Figure 1.

#### 2.2.2 Characterization and Insertion Studies on DMNs

The needle heights of the DMNs were visually assessed and recorded using a Leica EZ4 D digital microscope (Leica Microsystems, Milton Keynes, UK). Subsequently, mechanical investigations were conducted using a TA.XT2 Texture Analyzer (Stable Microsystems, Surrey, UK) in compression mode, formerly outlined by Donnelly et al.^30^ Parafilm M has been confirmed to replicate the thickness of excised skin for the insertion of MNs; therefore, it was folded into eight layers resulting in a thickness of ∼1 mm.^31^ The MN array was attached to a moveable probe of the Texture Analyzer and lowered onto the folded Parafilm M at a speed of 1.19 mm/s until a force of 32 N was exerted and held for 30 s. The MN array was removed from the Parafilm M, and the number of holes in each layer was counted. The needle heights were determined after insertion into the Parafilm M, and the percentage height reduction was calculated. When considering acceptable needle height reduction values for MN arrays, there are no regulatory standards or universal acceptance criteria available at present.^32^ For the purposes of this study, MNs were considered to possess suitable mechanical characteristics if the reduction in needle height was less than 10% and no significant difference in needle height was observed before and after insertion into Parafilm M. To establish that the DMNs could effectively penetrate a biological membrane, they were also inserted into excised full thickness neonatal porcine skin. An EX1301 optical coherence tomography (OCT) microscope (Michelson Diagnostics, Kent, UK) was used to observe the insertion properties of the DMN into skin.

#### 2.2.3 Determination of PRAS Content in DMNs

PRAS-containing DMNs were placed in glass vials containing 10 mL deionized water. Two magnetic stirring bars were added to the vials followed by mixing on a stirring plate until complete dissolution of the DMNs occurred. Subsequently, 10 mL ACN was added to the vial to precipitate the polymer. Samples were filtered using 0.2 μm Minisart syringe filters, diluted appropriately, and analyzed using reversed phase high-performance liquid chromatography (RP-HPLC) (method 1b outlined in Section 2.6).

### 2.3 Fabrication of Hydrogel-Forming Microneedles and Pramipexole Base-Containing Reservoirs

#### 2.3.1 Preparation of PRAB-Containing Directly Compressed Tablets

DCTs incorporating PRAB were prepared by mixing PRAB with various amounts of common oral tablet excipients including diluents (mannitol, sorbitol), binders (MCC, PVP), and super disintegrants (crospovidone). The precise compositions of PRAB-containing DCTs are outlined in Table 1. Tablet components were mixed together in their dry state using a pestle and mortar. A specific mass of each mixture was placed into a manual hydraulic press and 3 t (metric ton) of pressure was applied for 30 s to form the DCT. Following removal, fully formed DCTs were visually examined using a light microscope.

**Table 1:** Summary of the Formulations Explored for Fabricating PRAB-Containing DCTs

**formulation code**	**PRA base** (% w/w)	**excipients** (% w/w)					**theoretical tablet mass (mg)**
		**MCC**	**mannitol**	**PVP** 58 kDa	**sorbitol**	**crospovidone**	
**PB1**	100						50
**PB2**	100						30
**PB3**	20	30	50				100
**PB4**	10	30	20	20	10		100
**PB5**	20					80	100
**PB6**	20				80		100
**PB7**	20	30			50		100

#### 2.3.2 Physical Characterization of PRAB-Containing Directly Compressed Tablets

Initial physical characterization involved determining the uniformity of mass for DCT preparations. A protocol for this was adopted from the British Pharmacopoeia (BP),^33^ whereby 20 DCTs should be selected at random and weighed individually (*m*_*a*_). A mean mass was calculated (*m*_*b*_), and the percentage mass deviation of each DCT from the mean mass was calculated using eq 1. To achieve acceptable uniformity of mass, it is imperative that no more than two DCTs deviate from the mean by more than 7.5%.
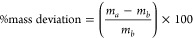
1

DCTs were also subjected to analysis using a tablet hardness apparatus, which measures their diameter, thickness, and hardness. The measurement of tablet harness entailed configuring the machine in compression mode, where a jaw approached the DCT, compressing the tablet between two rigid plates. This deliberate compression led to tablet fracture, enabling quantification of the fracture force. To determine diameter, thickness, and hardness, 10 DCTs were used.

#### 2.3.3 Dissolution Studies and PRAB Recovery from Directly Compressed Tablets

Dissolution/disintegration studies were conducted on DCT formulations that were fully formed when removed from the hydraulic press. A sample of four DCTs was selected from each formulation and placed in glass vials containing 10 mL PBS (pH 7.4). A magnetic stirring bar was added to the vials followed by mixing on a stirring plate at 500 rpm until complete dissolution/disintegration of the DCT occurred. The dissolution/disintegration time of each tablet was recorded. Subsequently, 10 mL ACN was added to the vial, and samples were filtered using 0.2 μm Minisart syringe filters and were diluted appropriately before analysis using RP-HPLC (method 1a outlined in Section 2.6).

#### 2.3.4 Fabrication of Hydrogel-Forming Microneedles

HFMNs were prepared by casting 500 mg of aqueous polymer blends into laser engineered micromolds containing 121 needles arranged in an 11 × 11 formation. The needles were conical in shape and 600 μm in height and had a base width of 300 and 300 μm interspacing. The polymer composition of each hydrogel formulation differed, and three formulations were investigated as detailed below.

##### 2.3.4.1 PVA/PVP Hydrogels

The PVA/PVP aqueous blend contained 15% w/w PVA (85–124 kDa), 10% w/w PVP (58 kDa), and 1.5% w/w citric acid.^34^ To obtain this, a 25% w/w stock solution of PVA and a separate 40% w/w stock solution of PVP were prepared with deionized water. In a Falcon tube, the required mass of citric acid was dissolved in a minimum volume of deionized water, and a specified mass of PVP stock was added followed by thorough mixing with a spatula. To this, the required amount of PVA was added and mixed to form a homogeneous blend. To remove bubbles, the mixture was centrifuged at 3500 rpm for 5 min. An aliquot of 500 mg was added to molds with subsequent centrifugation at 3500 rpm for 15 min and drying at ambient temperature for 48 h. The formed MNs were demolded, and side walls were removed using scissors. To induce chemical cross-linking by esterification, the MNs were placed on a glass Petri dish lined with baking paper and were heated at 130 °C for 3 h.

##### 2.3.4.2 “Normal Swelling” Gantrez Hydrogels

The “normal swelling” Gantrez aqueous blend contained 20% w/w Gantrez S-97 and 7.5% w/w PEG 10,000.^35^ In a Falcon tube, the required masses of Gantrez and PEG 10,000 were mixed together with deionized water to form a homogeneous blend. The mixture was then centrifuged at 3500 rpm for 5 min, and 500 mg was cast on to molds, followed by more centrifugation at 3500 rpm for 15 min. After drying at room temperature for 48 h, the MNs were demolded and cross-linked by esterification at 80 °C for 24 h.

##### 2.3.4.3 “Super Swelling” Gantrez Hydrogels

**Figure 2:**
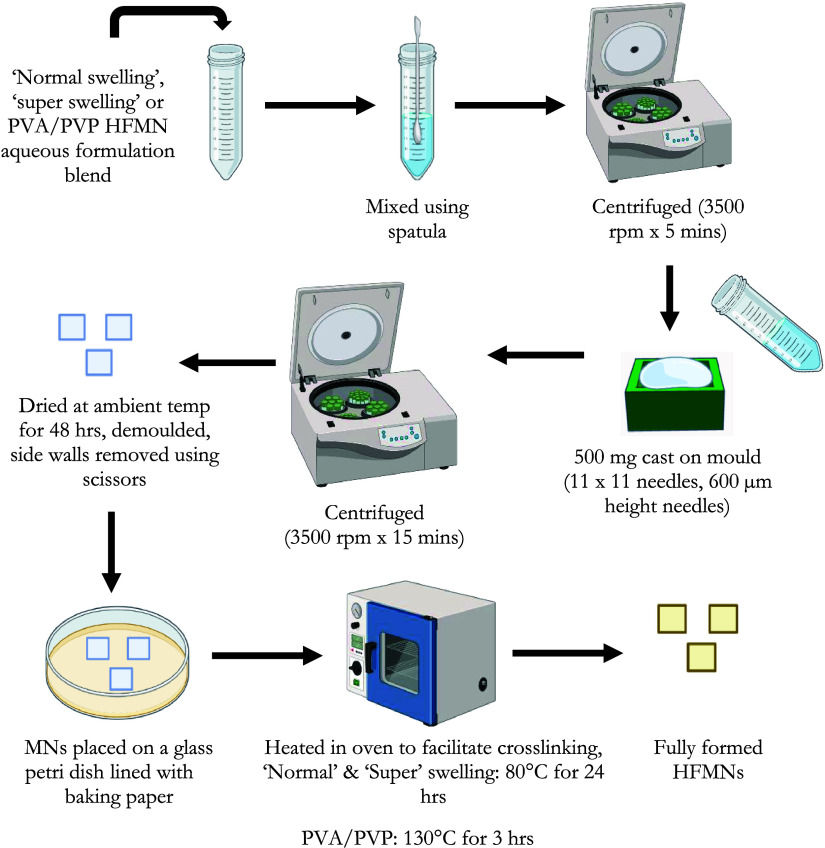
Diagrammatic representation of HFMN fabrication process.

The “super swelling” Gantrez aqueous blend contained 20% w/w Gantrez S-97, 7.5% w/w PEG 10,000, and 3% w/w sodium carbonate.^36^ Unlike the “normal swelling” formulation, this formulation contains sodium carbonate, which serves to reduce the cross-linking degree, resulting in a looser network structure. As a result, it exhibits a higher swelling capacity in comparison to the “normal swelling” formulation. In a Falcon tube, the required masses of Gantrez and PEG 10,000 were mixed together to form a homogeneous blend. Prior to the addition of sodium carbonate, it was prepared by grinding using a pestle and mortar for effective incorporation and to aid dissolution. Once a homogeneous blend was obtained, the mixture was centrifuged at 3500 rpm for 5 min. Again, an aliquot of 500 mg was added to molds with subsequent centrifugation at 3500 rpm for 15 min and drying at ambient temperature for 48 h. Following removal from molds, the esterification reaction was achieved in the same manner as “normal swelling” hydrogels by heating at 80 °C for 24 h. A summary of the preparation of HFMNs can be found in Figure 2.

#### 2.3.5 Preparation of Hydrogel-Forming Films

Hydrogel-forming films were prepared for use with side-by-side Franz cells to evaluate the permeation of PRA in solutions through a swollen hydrogel film. Hydrogel-forming films were fabricated using the same aqueous polymer blends that were used to fabricate HFMNs as outlined in Section 2.3.4. In the case of hydrogel-forming films, a 5g blend was poured on to a 5 × 3 cm flat Perspex base plate lined with a siliconized release liner on the surface and secured with stainless steel clamps. The blends were left to dry at room temperature for 48 h with subsequent removal of the film using a scalpel. The films were then removed, cut into 1 cm^2^ sections, and cross-linked using the same conditions as the HFMNs.

#### 2.3.6 Swelling Studies on Hydrogel-Forming Films

The swelling properties of each hydrogel formulation were assessed using the hydrogel-forming films that were prepared as outlined in Section 2.3.5. After cross-linking the films, the 1 cm^2^ segments were weighed individually in the dry state (*M*_0_). The films were then immersed in an excess of PBS (pH 7.4) and gradually absorbed fluid over time, causing the hydrogels to swell. At predefined time points, the hydrogel films were removed from PBS and dabbed with filter paper to remove excess fluid on the surface, and the mass of the swollen film was recorded (*M*_t_). The percentage swelling of each hydrogel film was determined using eq 2.
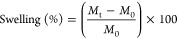
2

#### 2.3.7 Permeation of PRA through Swollen Hydrogel Films

**Figure 3:**
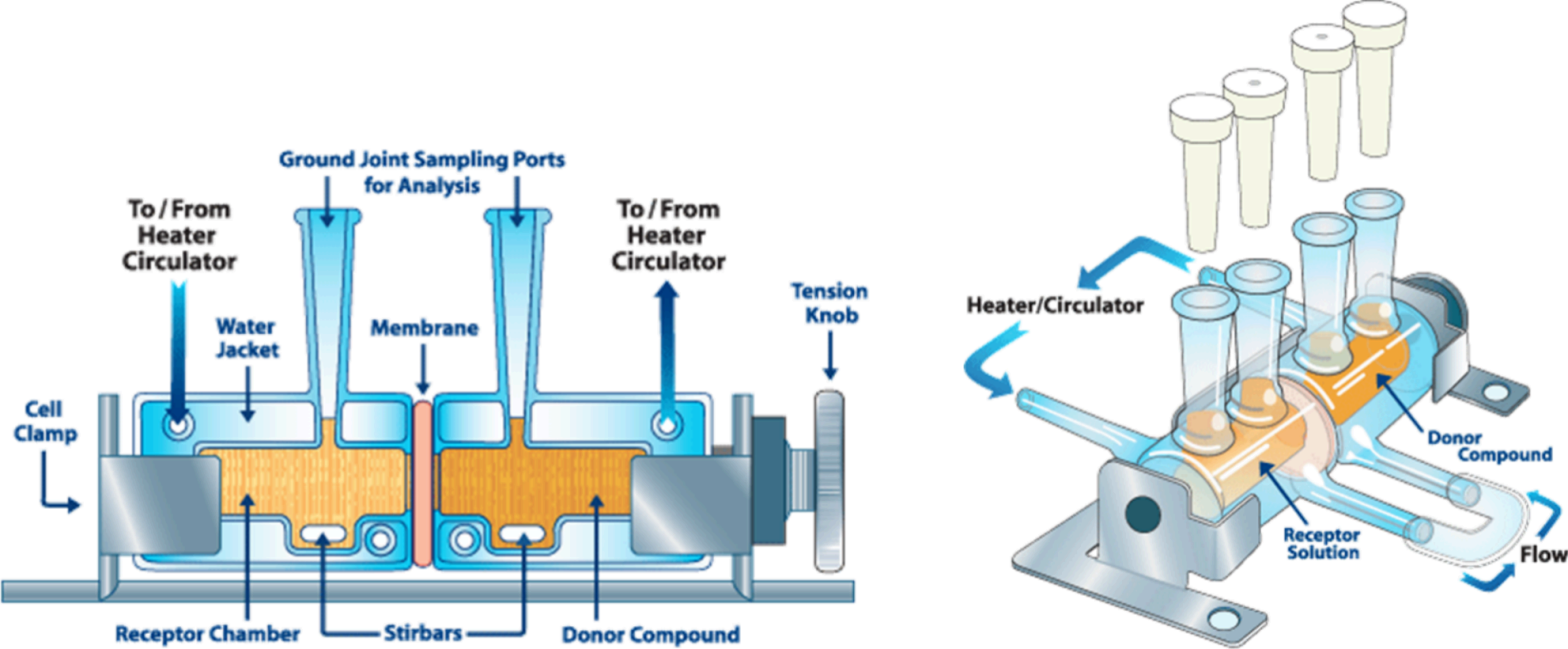
Illustration of side-by-side diffusion cell apparatus used to investigate solute diffusion across swollen hydrogel films.

Side-by-side diffusion cells were employed to ascertain the quantity of PRA that permeated through hydrogel films in their swollen state. Hydrogel-forming films prepared in Section 2.3.5 were used to conduct this experiment. The apparatus consisted of donor and receptor half-cell compartments and a water jacket that was maintained at 37 °C. A magnetic stirring bar (4 × 10 mm) was used to agitate the solution in both compartments at a speed of 600 rpm. Figure 3 depicts a schematic representation of the side-by-side diffusion cell apparatus designed to investigate solute diffusion across swollen hydrogel films.

Before the experiment commenced, the films were soaked in PBS (pH 7.4) for 24 h to establish equilibrium and prevent swelling during the course of the experiment. Subsequently, they were cut into a circular shape with a stainless-steel blade to effectively seal the gap between the donor and receptor compartments of the side-by-side diffusion cells. Following this, each circular film was placed between the two separate compartments and secured in place with Parafilm M to ensure that there was no leakage or evaporation of the release media. PRA was dissolved in PBS (pH 7.4) to obtain a 1 mg/mL solution (concentration expressed in terms of PRAB), and 3 mL was added to the donor compartment. Within the receptor compartment, 3 mL of freshly prepared PBS (pH 7.4) was introduced and maintained at 37 °C. At specified intervals of 1, 2, 3, 4, 5, 6, and 24 h, samples from the receptor compartment were collected and replaced with 3 mL of fresh PBS. Subsequently, all collected samples were subjected to analysis using RP-HPLC.

#### 2.3.8 Characterization of Hydrogel-Forming Microneedles

Characterization of HFMNs was conducted in the same manner as DMNs, as outlined in Section 2.2.2. Again, the percentage needle height reduction was calculated, and the number of holes created in each Parafilm M layer was determined.

### 2.4 Ex Vivo Permeation of Pramipexole from DMNs and HFMN-Reservoir Devices

Permeation of PRAS from DMNs across either dermatomed (350 μm) or full thickness (750 μm) neonatal porcine skin was investigated using Franz diffusion cells. Film or gel controls that did not contain MNs were also prepared. The controls contained the same quantity of drug as the DMN patch or PRAB-containing DCT to assess if the MNs were beneficial to drug delivery. Dermatomed skin was used to assess the permeation of PRAB from HFMN-reservoir devices. In this case, the control used was the reservoir alone, which was applied directly to the skin, without applying the HFMN.

The skin was obtained from stillborn piglets less than 24 h after birth using an electric dermatome (Integra Life Sciences, Padgett Instruments, NJ, USA) or a scalpel for full thickness skin, and hair was removed prior to the study. PBS (pH 7.4) was used as the receptor medium during this study as it mimics the ion concentration, osmolarity, and pH of human blood.^37^ PBS (pH 7.4) was placed in the receiver compartment of Franz cells along with a magnetic stirring bar and stirred at 600 rpm to enable equilibration, with the temperature maintained at 37 ± 1 °C. Skin was fixed to the donor compartment of the Franz cells using cyanoacrylate glue. The DMNs were inserted into the skin using manual pressure for 30 s, and the donor was carefully clamped to the receiver compartment, ensuring that no air bubbles were present. PVA/PVP HFMNs were also inserted into the skin using manual thumb pressure for 30 s, after which 20 μL of water was placed on top of the MN surface to enhance the adhesion of the reservoir. A stainless-steel weight was placed on top of the MNs to keep them in place. To prevent evaporation, Parafilm M was used to seal the sampling arm and top of the donor compartment. At predefined intervals, 200 μL samples were removed from the sampling arm, diluted appropriately with PBS (pH 7.4), and analyzed using RP-HPLC. An equal volume of prewarmed PBS (pH 7.4) was added to the receiver compartment each time.

### 2.5 In Vivo Delivery of Pramipexole

#### 2.5.1 In Vivo Study Design

Approval for this study was granted by the Committee overseeing the Biological Services Unit at Queen’s University Belfast. The research procedures were carried out in compliance with Procedure Project License number 2903 and Procedure Individual License numbers 2198, 2059, and 2127, adhering to the guidelines outlined by the Federation of European Laboratory Animal Science Associations and the European Convention for the protection of vertebrate animals used for experimental and other scientific purposes. These procedures were executed with commitment to uphold the principles of the 3Rs, which stand for replacement, reduction, and refinement, in animal research. Female Sprague–Dawley rats (*n* = 22) aged 10–12 weeks with a weight of 252 ± 23 g were used in this study and were acclimatized to the animal house conditions for 7 days prior to study commencement.^38^ The rats were separated into three cohorts. Cohort 1 (*n* = 6) received a 35.76 mg/kg dose of PRAS by oral gavage (equivalent to 25 mg/kg PRAB and 6.25 mg PRAB per rat). The oral solution was prepared by dissolving PRAS in deionized water. The bioavailability of orally administered PRA has been reported to be 90%, which would result in approximately 5.63 mg PRAB reaching systemic circulation.^39^ Cohort 2 (*n* = 8) received PRAS via application of one DMN patch. Specifically, 21.46 mg PRAS (equivalent to 15.01 mg PRAB) was loaded into each MN patch. According to previous studies on DMNs, the bioavailability of the DMN was expected to be in the range of 35–45%; thus, the total dose that would be received by each rat was predicted to be 5.25–6.75 mg PRAB.^40^ Cohort 3 (*n* = 8) received two PRAB loaded HFMN-DCT devices. Specifically, 19.05 mg of PRAB was loaded into each DCT (38.1 mg in two DCTs). The bioavailability of HFMN-devices was expected to be in the range of 10–20%, based on previous studies.^41^ In consideration of this, approximately 3.81–7.62 mg would potentially be delivered from the HFMN-DCT devices. Because of the potential overlapping of delivered PRA in each cohort, the oral, DMN, and HFMN cohorts could be compared in terms of pharmacokinetic parameters. For both the DMN and HFMN cohorts, the patches were applied to the back of each animal at the start of the study. As there were eight rats in each MN cohort, patches were removed from four rats in each cohort after 24 h, and the patches on the remaining four rats in each cohort were kept in place for a duration of 5 days. A summary of the doses administered to rats in each cohort can be found in Table 2, with all values given in terms of PRAB.

**Table 2:** Summary of Doses Administered to Animals in Each Cohort and Expected Bioavailability

**cohort**	**PRA administration method**	**dose administered (mg/rat)**	**dose administered**(mg/kg)	**expected bioavailability (%)**	**expected PRA delivered (mg)**
1	oral gavage	6.25	25	90	5.63
2	1× DMN	15.01	60.04	35–40	5.25–6.75
3	2× HFMNs	38.1	152.4	10–20	3.81–7.62

Hair was removed from around the area of MN application for cohort 2 and cohort 3 prior to patch application. To do this, 1 day before MN application, rats were sedated using gaseous anesthetic (2–4% v/v isoflurane in oxygen), and hair was shaved using electric clippers. Following this, hair removal cream was applied to remove any remaining hair in the area. To ensure complete restoration of skin barrier function, rats were left for 24 h before patch application.^42^

**Figure 4:**
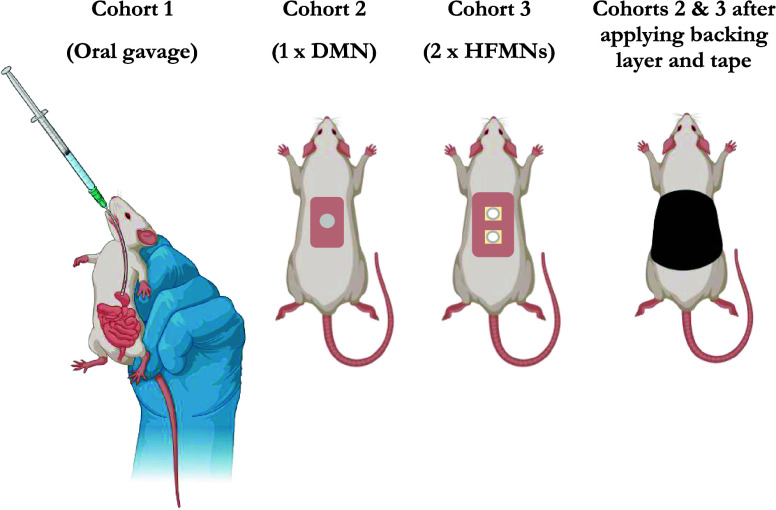
Summary of PRA delivery route to each cohort of rats. Created using BioRender.com.

The next day, rats were sedated again using gaseous anesthetic, and MN patches were applied to the shaved area on their backs using finger pressure for 30 s to facilitate insertion of MNs into the skin. Following the application of DMNs, the appropriate backing layer was affixed in place. In the case of HFMNs, 20 μL of water was added on top of the HFMN, and a DCT was placed on the surface. Once more, the suitable backing layer was applied to ensure that the HFMN-device remained in place. To further ensure that the MN patches were secured in position for the intended duration, kinesiology tape (Proworks Corporation, Corvallis, Oregon, United States) was gently wrapped around the backs and abdomens of rats in cohorts 2 and 3. Figure 4 provides a visual summary detailing the delivery route of PRA to each cohort of rats. Blood samples were acquired through tail vein bleeds at predefined time points over a 5 day period. Approximately 200 μL of blood was collected at each time point into preheparinized 1.5 mL Eppendorf tubes. Upon completion of the study at day 5, animals were humanely sacrificed using carbon dioxide asphyxiation, and any remaining blood was harvested through cardiac puncture.

Following the blood collection process, samples were centrifuged at 2000*g* for 5 min at 4 °C to separate plasma from blood. Subsequently, the plasma fraction was collected, and PRA was extracted from plasma by employing a liquid–liquid extraction technique. In brief, 200 μL of methyl *tert*-butyl ether (MTBE) was added to an Eppendorf tube containing 45 μL of PRA-containing plasma and 5 μL 1 M sodium hydroxide (NaOH).^43,44^ Each Eppendorf tube was vortexed at 1500 rpm for 15 min at 20 °C followed by centrifugation at 13,800 rpm (17,458*g*) for 10 min at 4 °C. The resulting supernatant (150 μL) was transferred to a glass culture tube and subjected to a drying process under a stream of nitrogen at 40 °C for 40 min using a Zymark TurboVap LV Evaporator Workstation. Following this, reconstitution was carried out with 50 μL 5:95% v/v water/methanol. The glass culture tubes were sonicated for 30 s and vortexed for 30 s. The samples were then transferred to Eppendorf tubes and centrifuged at 13,800 rpm (17,458*g*) for 10 min at 4 °C before being placed in HPLC vials containing 150 μL glass inserts with polymeric feet for analysis using UPLC-MS/MS.

#### 2.5.2 PRAB Remaining in Directly Compressed Tablet after Hydrogel-Forming Microneedle Removal

Following 24 h of HFMN application, four rats within cohort 3 had their patches removed, whereas the remaining four rats had HFMNs removed on day 5 of the study. Each rat had two HFMNs and two DCTs. For each individual rat, the residual DCTs and backing layers were placed in glass vials containing 10 mL deionized water and 10 mL ACN and vortexed to dissolve any remaining PRAB. Similarly, the remaining HFMN patches were cut into small sections and also placed in a glass vial containing 10 mL deionized water and 10 mL ACN. Once more, thorough vortexing was conducted to ensure the dissolution of any PRAB present. The resultant samples were then filtered, centrifuged at 13,800 rpm (17,458*g*) for 10 min, and diluted appropriately prior to HPLC-UV analysis.

### 2.6 Pharmaceutical Analysis of Pramipexole

In vitro and ex vivo analysis of PRA was performed using a reversed phase HPLC Agilent 1200 system coupled with a UV detector. Chromatography was carried out on a Phenomenex InertClone ODS(3) C18 column (250 × 4.6 mm internal diameter, 5 μm packing), with UV detection conducted at 265 nm. Numerous methods were developed, with the solvent for drug dissolution choice and mobile phase ratio being the main differing factors. For each method, PRAB and PRAS were validated separately, resulting in a total of four validated in vitro methods. A summary of the chromatographic conditions for each method can be found in Table 3. The aqueous components of mobile phases were placed in a sonicator to aid degassing preceding their use in HPLC, and the Agilent Chemstation Software was used for chromatographic analysis.

**Table 3:** Chromatographic Conditions of HPLC-UV Methods to Detect PRA following In Vitro and Ex Vivo Studies

	**method 1a**	**method 1b**	**method 2a**	**method 2b**
PRA form	base	salt	base	salt
dissolution solvent	ACN and water (50:50% v/v)	ACN and water (50:50% v/v)	PBS (pH 7.4)	PBS (pH 7.4)
mobile phase	0.1% TFA in water and ACN (75:25% v/v)	0.1% TFA in water and ACN (75:25% v/v)	0.1% TFA in water and ACN (90:10% v/v)	0.1% TFA in water and ACN (90:10% v/v)
flow rate(mL/min)	0.6	0.6	1	1
retention time (min)	3.91	3.91	4.17	4.17
injection volume (μL)	40	40	40	40
UV detection (nm)	265	265	265	265
total run time (min)	6	6	6	6

Once dissolved, PRAS dissociates into PRAB; therefore, to save time and resources, a bioanalytical method was validated to quantify PRAB only. Analysis of PRAB in rat plasma was conducted using an Acquity UPLC i-Class system coupled with a Xevo TQ-MS (triple quadrupole MS/MS) mass spectrometer (Waters, Manchester, UK). The system was used in electrospray positive mode (ESI^+^) with a precursor ion of 212.0 (*m*/*z*) and fragment ions of *m*/*z* 126.05 and *m*/*z* 153.1, with a collision energy of 25 eV used to generate both fragment ions. The nebulizer gas was 30 psi, ion transfer capillary temperature was 250 °C, dwell time was 75 ms, and gas flow was 8 L/min. A Waters Atlantis dC18 column (3.9 × 150 mm internal diameter, 3 μm packing) was used to achieve separation. The isocratic method incorporated a mobile phase containing 60:40% v/v water/formic acid (0.1%) and MeOH/formic acid (0.1%) at a flow rate of 0.5 mL/min. The column and sample temperatures were maintained at 40 and 12 °C, respectively. After a sample injection volume of 5 μL, a peak was detected at 1.89 min, resulting in a total run time of 3 min. The MassLynx software was used to process data, and peak areas were used to determine PRA concentration.

The in vitro methods were validated following guidelines outlined by the ICH of Technical Requirements for Registration of Pharmaceuticals for Human use, Validation of Analytical Procedures Q2 (R1) 2005.^45^ The in vivo bioanalytical method was validated following Bioanalytical Method Validation M10 guidelines.^46^ The validation characteristics that were examined were specificity, accuracy, precision, linearity, range, limit of detection (LoD), limit of quantification (LoQ), and lower limit of quantification (LLOQ) for the in vivo method. Specificity was analyzed by injecting a blank solvent sample for in vitro methods or a blank rat plasma sample following the extraction procedure for the in vivo method. The blank samples were then spiked with PRA and analyzed again to ensure that PRA could be separated from other components present. To evaluate linearity and range, each calibration curve consisted of at least five calibration standards for the in vitro methods and at least six standards for the in vivo method, and linearity was established over 3 days. Range is denoted as the interval between the upper and lower concentration of analyte that demonstrates a suitable level of linearity. The accuracy of an analytical procedure relates to the closeness of agreement between an accepted reference value or true value and the value found.^45^ To establish accuracy for the in vitro methods, three concentrations were assessed, representing low, medium, and high concentrations within the calibration curve range. For the in vivo method, four quality control (QC) samples were assessed including LLOQ, low QC, medium QC, and high QC. Inter- and intraday accuracy was assessed in each validated method. Interday accuracy was determined between consecutive days, whereas intraday accuracy was calculated using samples within 1 day. Verification of accuracy was achieved by attaining a recovery percentage within the range of ±20% for LLOQ and ±15% of the expected concentrations chosen for analysis. The precision of an analytical procedure is interpreted as the closeness of agreement between a series of measurements obtained from multiple sampling of the same sample under the defined conditions. Precision is expressed as the variance, standard deviation, or coefficient of variation of a series of measurements.^45^ For each method, interday precision was determined by analyzing concentrations on three different days, and intraday precision was investigated using concentrations prepared three separate times within 1 day. The concentrations used to determine accuracy were also the concentrations used to calculate precision. The lowest amount of analyte in a sample that can be detected but not necessarily quantified as an exact value is known as the detection limit of an analytical procedure. The LoQ is defined by the ICH as the lowest amount of analyte in a sample that can be quantitatively determined with suitable precision and accuracy.^45^ In the realm of bioanalytical methodology, the LLOQ is described as the lowest concentration of analyte in a sample that can be quantified reliably, with an acceptable accuracy and precision. The LLOQ was established by identifying the lowest PRA concentration measured where the observed percentage recovery remained within 20% of the anticipated concentration value.

### 2.7 Pharmacokinetic Analysis

Following pharmaceutical analysis, pharmacokinetic parameters of the delivery profiles obtained for each cohort were determined. The maximum plasma concentration (*C*_max_) and the time taken to achieve *C*_max_ (*T*_max_) were obtained by inspecting the raw data. The total PRA exposure, denoted by area under the curve (AUC), from each delivery profile was also determined using the linear trapezoidal method from the beginning of the experiment at time zero (*t* = 0) to the last experimental time point at 5 days (*t* = 120 h). The AUC between these two time intervals (*t*_2_ – *t*_1_) and their corresponding concentrations (*C*_1_ + *C*_2_) was calculated using eq 3.
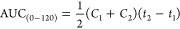
3

### 2.8 Statistical Analysis

Statistical analysis was performed using GraphPad Prism version 9.0 (GraphPad Software, San Diego, California, USA). For parametric data, an unpaired *t* test was conducted to assess two groups, whereas one-way analysis of variance (ANOVA) was used to analyze the differences between various groups. All data were expressed as means ± standard deviation. Statistical difference was denoted by *p* < 0.05 in all cases.

## 3 Results and Discussion

### 3.1 Pharmaceutical Analysis of Pramipexole

The experiments conducted here involved formulating PRA-containing MN patches and reservoirs that contained hydrophilic polymers. To measure drug content within these patches and reservoirs and to evaluate their performance in *ex vivo* studies, HPLC analysis was crucial. Prior to injecting a sample into HPLC, water-soluble polymers must be removed to prevent polymer precipitation upon contact with the organic portion of the mobile phase. To achieve this, ACN was utilized to induce polymer precipitation. As a result, two methods, one for PRA base (**method 1a**) and one for PRA salt (**method 1b**), were validated that involved dissolving PRA in a mixture of 50% ACN and 50% water. Another method was designed to facilitate the analysis of Franz cell samples, which would contain drug dissolved in PBS (pH 7.4). Consequently, samples for the validated base (**method 2a**) and salt (**method 2b**) methods were dissolved in PBS. A sensitive method was required to analyze rat plasma samples. HPLC coupled with a UV detector lacked sufficient sensitivity; therefore, it was necessary to use a UPLC system in conjunction with a triple quadrupole MS/MS mass spectrometer detector.

Specificity of the methods was demonstrated as PRA could be successfully identified in the presence of possible interferences. The correlation coefficients for each method were ≥0.9996. Details of the calibration graph characteristics, encompassing the slope, *y*-intercept, LoD, and LoQ, are provided in Tables 4 and5.

**Table 4:** Calibration Parameters for Validated Methods to Be Used during In Vitro Experiments, Expressed through Linear Regression with R2, LoD, and LoQ

**method**	**PRA form**	**range** (μg/mL)	**slope**	*y*-intercept	***R***^**2**^	**LoD** (μg/mL)	**LoQ** (μg/mL)
**method 1a**	base	0–100	154.52	–3.9041	1	0.25	0.75
**method 1b**	salt	0–100	147.01	–4.7327	1	0.17	0.50
**method 2a**	base	0–100	86.848	14.592	1	0.76	2.29
**method 2b**	salt	0–100	87.439	20.225	1	0.78	2.35

**Table 5:** Calibration Parameters for Validated Methods to Be Used during In Vivo Experiments, Expressed through Linear Regression with R2, LoD, and LLoQ

**method**	**PRA form**	**range** (ng/mL)	**slope**	*y*-intercept	***R***^**2**^	**LoD** (ng/mL)	**LLoQ** (ng/mL)
**method 3**	base	0–50	4689.5	2206.6	0.9996	0.89	1
**method 3**	base	75–1000	4557.2	–30867	0.9998	18.53	75

All of the methods were deemed accurate as a percentage recovery between 98% and 103% was obtained for all methods. For all methods, the coefficient of variance was below 9%, rendering the methods suitable for their intended purpose. As a result, the validated methods could be used with confidence to analyze samples obtained from forthcoming studies, as detailed in this paper.

### 3.2 Dissolving Microneedles

#### 3.2.1 Characterization and Insertion Studies on Dissolving Microneedles

PVP was used to formulate PRAS-containing DMNs as it hardens well on drying and exhibits excellent mechanical strength as a result of its chemical structure, which features a rigid ring.^13,47^ The monomer unit of PVP contributes to intermolecular stiffness, which further enhances its mechanical strength. The mechanical properties of PVP depend on both its concentration and molecular weight. Previous research has proven that MNs comprising PVP with a molecular weight equal to, or exceeding, 9000–10,000 Da can exert sufficient strength to penetrate skin after applying average thumb pressure.^48^ When used alone, PVP can display brittle properties, and it is common to add a plasticizer such as PVA, polyethylene glycol (PEG), or glycerol to the matrix. The amide groups present in PVP have a basic nature that enables them to readily accept protons; therefore, hydrogen bonding occurs between the carbonyl oxygens of PVP and hydroxyl groups of the plasticizer, which further increases the mechanical properties of the MNs.^49^ Furthermore, it has been reported that these noncovalent interactions will lead to an increase in free volume within the matrix promoting water uptake into the material, ultimately resulting in a faster dissolution time in an aqueous environment.^50^ Of course, when drug is added to the polymer matrix, the MN insertion properties may be altered. The effect of altering PRAS concentration and polymer composition on the mechanical properties of DMNs was investigated here.

Various PRAS-containing DMNs were formulated and are presented in Table 6. After visual inspection using a light microscope, SD4, SD5, SD6, and SD7 were all chosen to undergo further characterization tests as they appeared to have fully formed needles upon removal from molds.

**Table 6:**
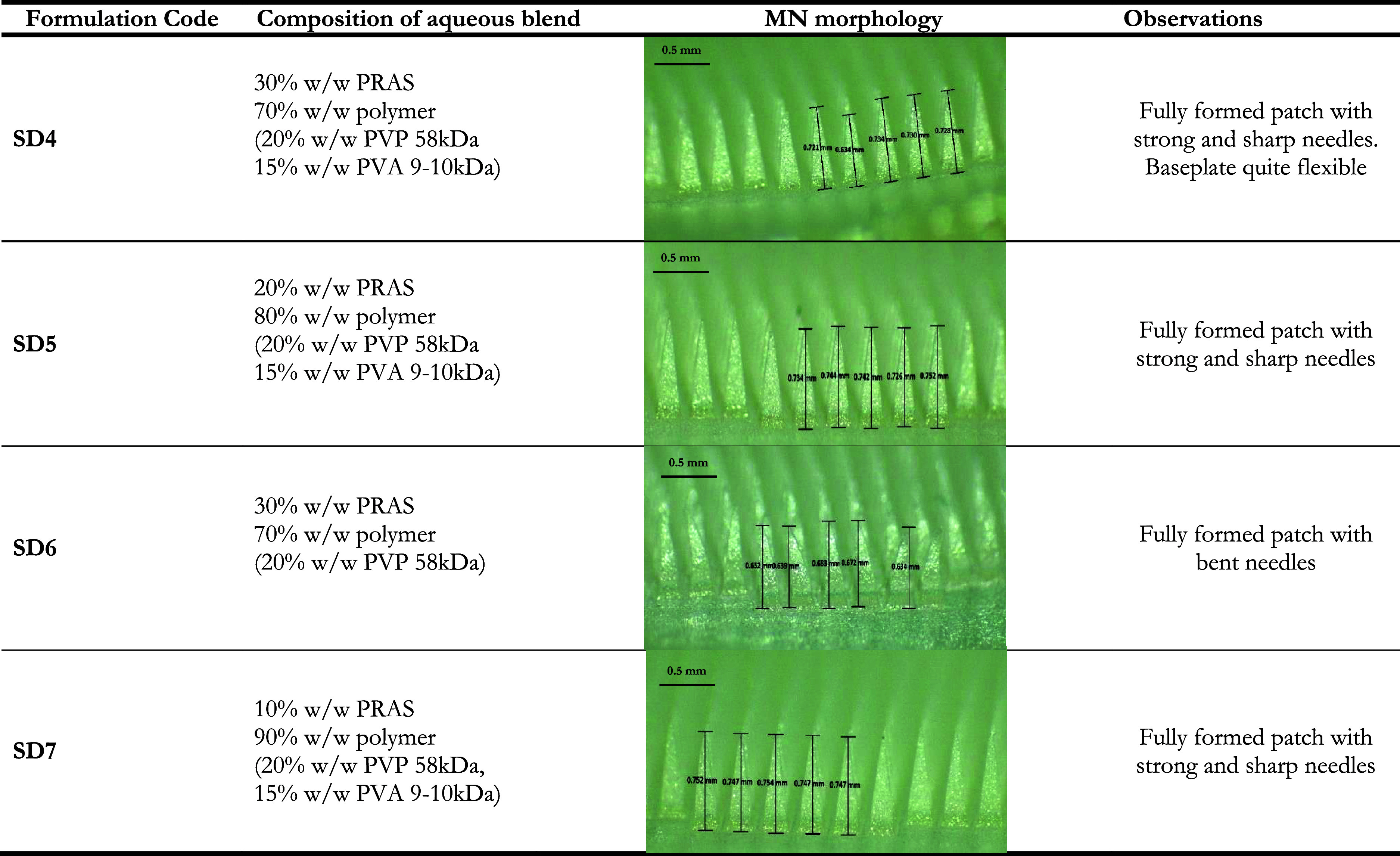
Formulations of Aqueous Blends Investigated for the Fabrication of PRAS-Containing DMNs

**Figure 5:**
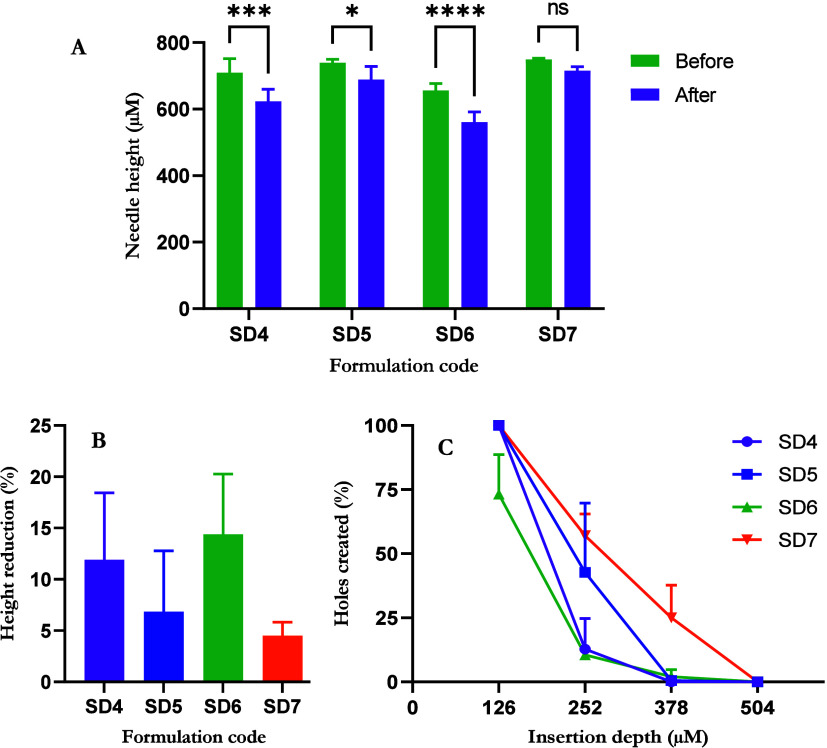
(A) PRAS-containing DMN needle heights before and after the insertion test (means + S.D., *n* = 4), (B) percentage height reduction of needles after insertion into Parafilm M (means + S.D., *n* = 4), and (C) percentage insertion profile of DMNs into Parafilm M (means + S.D., *n* = 4).

SD4 and SD6 both contained 30% w/w PRAS, with SD4 containing both PVA and PVP, whereas SD6 only contained the polymer PVP. Before insertion, SD6 needles had an average height of 656 ± 2.06 μm. Considering that the molds used had needle heights of 750 μm, the needles did not form properly and were evidently bent as depicted in Table 6. Additionally, the needle height reduction after insertion into Parafilm M was significant at 14.38 ± 5.87%, as shown in Figure 5B (*p* < 0.0001). Before insertion, the mean needle height of the SD4 formulation was 709.4 ± 42.41 μm, showing better formed needles than SD6. However, SD4 also had a significant needle height reduction of 11.91 ± 6.51% (*p* = 0.0001). Overall, it was evident that 30% w/w PRAS resulted in needles exhibiting poor mechanical properties, and statistical analysis indicated that there were no significant differences between SD4 and SD6 in terms of percentage height reduction (*p* = 0.5467).

Decreasing the drug loading to 20% w/w of the aqueous blend in SD5 resulted in a more promising formulation with mean initial needle heights of 739.6 ± 9.94 μm displayed in Figure 5A. However, a significant height reduction of 6.83 ± 5.95% was observed after the insertion test (*p* = 0.0293). SD7 illustrated the strongest drug containing needles with an initial needle height of 749.4 ± 3.36 μm. The needles of this 10% w/w PRAS formulation reduced by 4.51 ± 1.30% after the insertion test. This was a very encouraging result, and there was no statistically significant difference in needle height before and after insertion into Parafilm M (*p* = 0.2392).

Parafilm M was used to simulate skin with each layer possessing a thickness of 126 ± 7 μm.^31^ Insertion study results presented in Figure 5C highlighted that all but one DMN formulation (SD6) effectively accomplished 100% penetration through layer 1 of Parafilm M after a mean force of 32 N was applied. SD4, SD5, and SD7 were capable of penetrating the third layer of Parafilm M, equating to a skin depth of 378 μM. This represents approximately 50% of the needle height and confirms that these formulations would have sufficient strength to effectively overcome the barrier properties of the stratum corneum.

Formulation SD7 was taken forward for analysis of drug content and ex vivo permeation performance as it inserted well into the simulated skin model with minimal needle height reduction. It is important to mention that the force used to insert the MN arrays was selected based on previous studies that evaluated average force applied by human volunteers. There are a wide variety of studies highlighting that MN arrays can be self-applied successfully by patients.^25,51^ However, in the current study, PRA-loaded MN arrays are aimed for Parkinson’s disease patients, which could present limitations when applying the patches. In this case, a potential applicator device might be useful to ensure consistent MN application. However, this aspect is beyond the scope of the current work.

**Figure 6:**
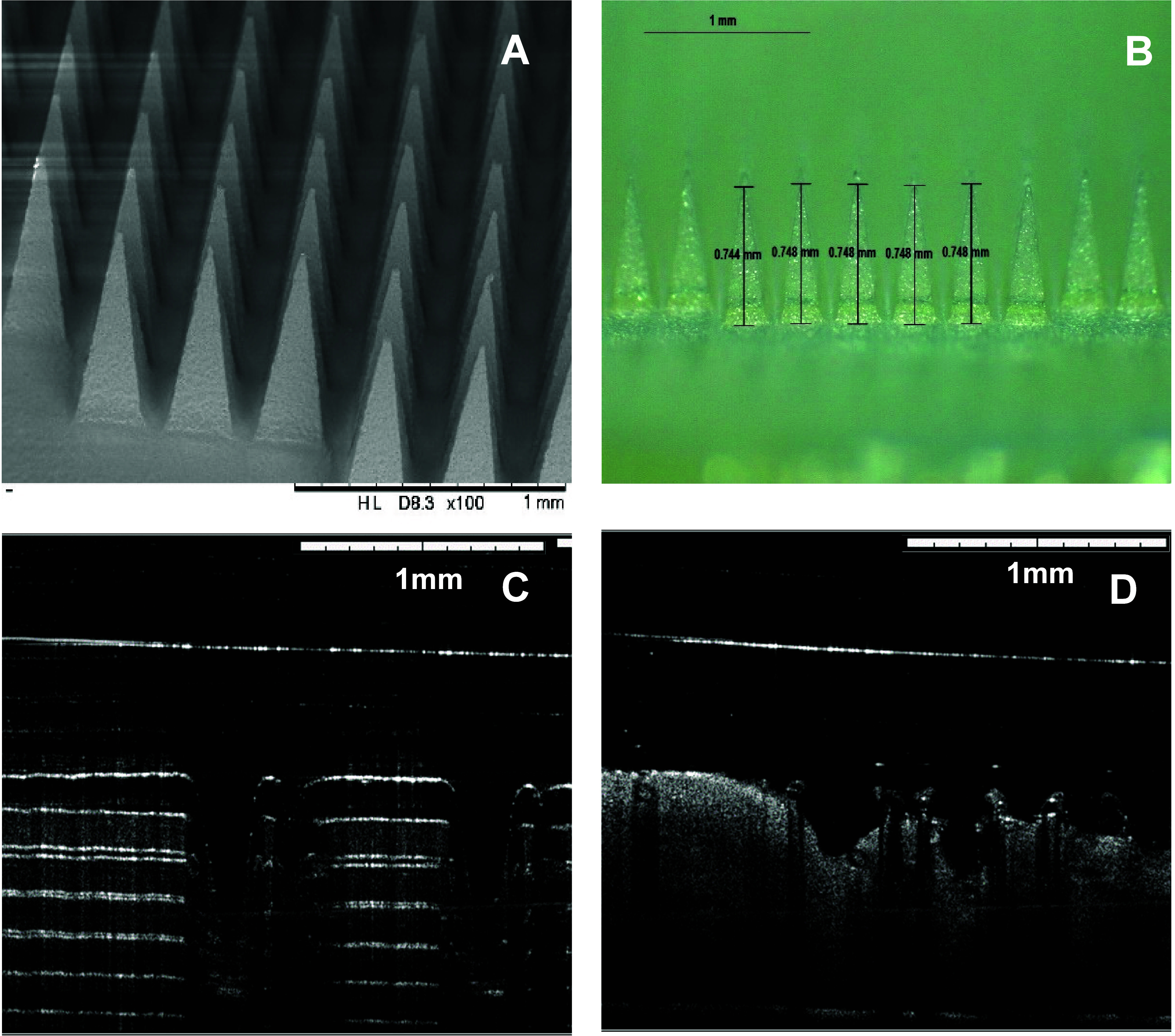
(A) Scanning electron microscope image and (B) digital light microscope image of SD7 DMNs. Optical coherence tomography image of SD7 DMN inserted into (C) Parafilm M and (D) neonatal porcine skin.

Following the results of the mechanical investigations, needles of formulation SD7 were viewed under the SEM and optical microscopy (Figure 6A,B). These images show that the needles present a homogeneous appearance and that no obvious drug accumulation can be seen. These images indicate that the drug is dispersed within the polymer matrix. To ascertain insertion/dissolution of the needles, OCT was also used to view the needles inserted into eight layers of Parafilm M and full thickness neonatal porcine skin (Figure 6C,D). It can be seen that the MNs can be successfully inserted through at least the first three layers of Parafilm M. These results are consistent with the insertion study presented in Figure 5C. Finally, OCT insertion into full thickness revealed that the needles started the dissolution process just after insertion. Figure 6D shows how the needles began to dissolve, losing their sharp needle tip shape right after insertion.

#### 3.2.2 Determination of PRAS Content in Dissolving Microneedles

It was important to determine the drug content within each DMN as PRAS was distributed across the needle tips and the baseplate. A needle dense mold (600 needles per patch) was chosen for preparation of DMNs to maximize drug loading. To determine PRAS content within the DMNs, the most promising formulation (SD7) was dissolved, and drug content was analyzed using RP-HPLC. Formulation SD7 had a drug loading of 15.01 ± 2.53 mg, expressed in terms of PRAB.

#### 3.2.3 Ex Vivo Permeation of PRAS from Dissolving Microneedles

**Figure 7:**
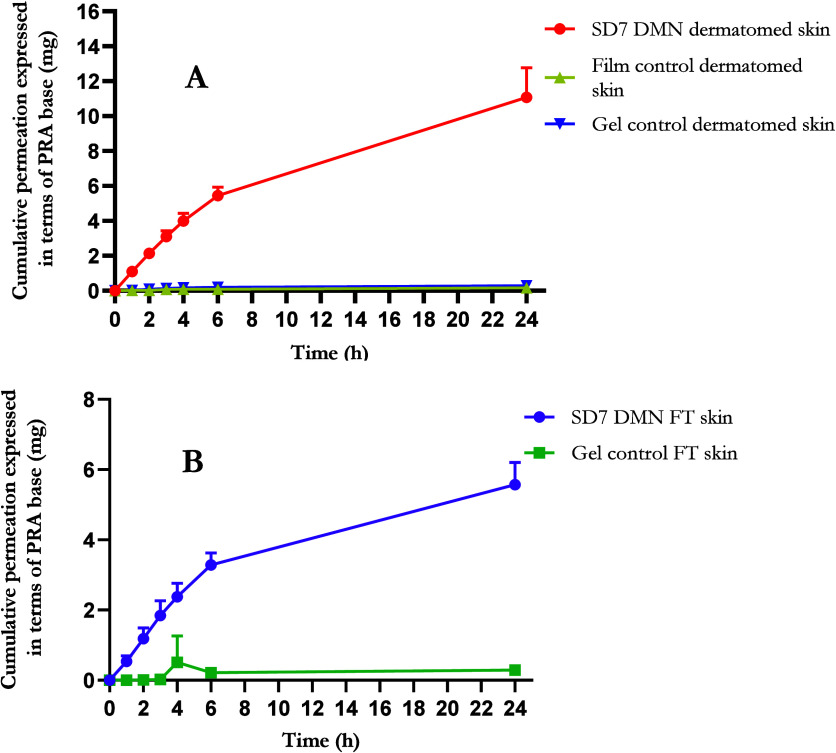
Ex vivo permeation profile of PRAS-containing DMNs (SD7) and controls through (A) dermatomed skin and (B) full thickness skin (FT represents full thickness neonatal porcine skin) (means + S.D., *n* = 5).

The ex vivo permeation of PRAS from DMNs across dermatomed and full thickness neonatal porcine skin was evaluated using the Franz cell apparatus. Initially, PRAS delivery was assessed across dermatomed neonatal porcine skin (thickness 350 μm) as it has been reported to be a more accurate representation of transdermal drug delivery (TDD) in vivo in comparison to full thickness skin (thickness 750 μm).^52^ Most drugs are absorbed through the dermal microvasculature, which is located at a distance of approximately 200–400 μm from the stratum corneum in living skin. Full thickness skin adds an additional barrier to TDD that is not a true portrayal of the in vivo structure of skin, as it increases the distance between the DMN on the skin surface and its interface with the receptor medium and an active microcirculation is absent, impeding drug permeation. Nonetheless, it remains an important skin model, particularly for intradermal deposition of drugs. Following 24 h of the ex vivo permeation study across dermatomed skin, the PRAS-containing DMN (SD7) delivered 11.07 ± 1.69 mg, representing 73.81 ± 11.28% of the total drug content within the DMN, as presented in Figure 7A.

In the first instance with PRAS-containing DMNs, a film control was prepared, which contained the same polymer formulation and drug content as the DMN patch. After 24 h of the Franz cell experiment, 0.16 ± 0.01 mg was delivered across the dermatomed skin, equating to a mere 1.07 ± 0.10% of the total drug within the film that was significantly less than that delivered from the DMN (*p* < 0.0001). When the apparatus was dismantled after 24 h, it was evident that the majority of the film was intact and had not dissolved, resulting in low PRA permeation. As PRA has ideal properties for TDD, the low permeation may have been due to the lack of water present in the film, hindering its dissolution. It was therefore considered appropriate to assess the permeation of PRA from a gel control instead. Similarly, the gel control was prepared using the same formulation and drug content as the DMN. The gel was freshly prepared before commencement of the experiment to ensure that it did not dry. A significant increase in permeation was observed from the PRAS-containing gel control (0.29 ± 0.03 mg delivered, representing 1.92 ± 0.22% of loaded drug) compared to the film as almost double was detected in the receiver compartment after 24 h (*p* = 0.0377). Consequently, gel controls were preferred over film controls.

It was evident from insertion studies into the simulated skin model, Parafilm M, that formulation SD7 was capable of piercing the third layer of Parafilm M, representing an insertion depth of 378 μm. Although the majority of drug resides in the baseplate and the proportion of needles reaching the third layer was small (25 ± 12.73% of needles in SD7), given that the thickness of dermatome skin was approximately 350 μm, some of the needles may have contacted the receptor compartment media immediately, resulting in prompt dissolution. Consequently, it was important to investigate delivery through full thickness skin. After 24 h, 5.57 ± 0.63 mg was delivered through the full thickness skin into the receiver compartment, equating to 37.12 ± 4.23% compared to 0.22 ± 0.02 mg (1.44 ± 0.12% of total drug) from the PRAS gel control, as shown in Figure 7B. It was expected that delivery would be lower through full thickness skin; however, this is still a substantial delivery efficiency, further confirming that the MN shafts were successful in creating microchannels for drug diffusion. Once again, delivery from the DMN exceeded the gel control.

PRA has been formulated in alternative transdermal drug delivery systems such as conventional patches and different types of MN patches.^53−55^ Considering that the MN patches described in this article had a surface of around 1 cm^2^, the results can be compared with the results reported in these previously published studies. Conventional transdermal patches were capable of administering up to 0.8 mg/cm^2^ of PRA after 24 h. On the other hand, Hoang et al. described that the application of a PRA solution on the surface of the skin after the application of solid MNs led to a permeation of up to 1.6 mg/cm^2^ of the drug after 12 h.^54^ It is obvious that creating channels for drug diffusion will lead to a higher drug permeation. Alternatively, Saepang et al. prepared dissolving MN arrays for PRA delivery, achieving permeations of up to 2 mg/cm^2^ after 24 h. However, the permeation achieved in the present study was superior. This could be due to the higher drug loading achieved in the present study.

### 3.3 Hydrogel-Forming Microneedles

#### 3.3.1 Swelling Properties of Hydrogel-Forming Films

**Figure 8:**
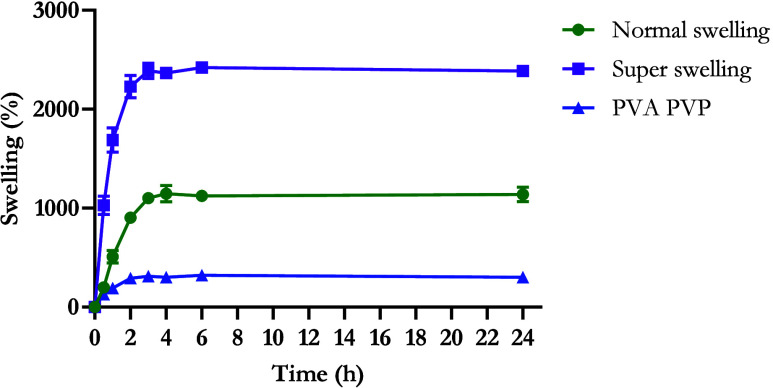
Swelling profiles of PVA/PVP and “normal swelling” and “super swelling” Gantrez hydrogel films in PBS (pH 7.4) over 24 h (means ± S.D., *n* = 5).

The swelling properties of PVA/PVP and “normal swelling” and “super swelling” Gantrez hydrogel-forming films in PBS (pH 7.4) was investigated with swelling profiles displayed in Figure 8 After 24 h, the “super swelling” Gantrez hydrogel-forming films had swollen by 2385.18 ± 16.45% compared to 1137.98 ± 72.19% for “normal swelling” Gantrez and 302.01 ± 17.72% for PVA/PVP films. The “super swelling” formulation exhibited a significantly higher swelling capacity than the other formulations, swelling twice as much as the “normal swelling” films (*p* < 0.0001) and almost eight times that of the PVA/PVP films (*p* < 0.0001) over a period of 24 h.

Hydrogels undergo a state of swelling due to intermolecular interactions occurring between water molecules and hydrophilic moieties located within polymer chains when exposed to an aqueous environment, whereas their resistance to dissolution is attributed to the established cross-links between polymer chains.^56−58^ The swelling behavior is influenced significantly by the cross-linking ratio, which is defined as the ratio of cross-linking agent moles to the moles of polymer repeating units. A higher cross-linking ratio results in increased integration of the cross-linking agent into the hydrogel structure, leading to a more compact configuration and ultimately less swelling. The chemical composition of polymers also affects swelling, with hydrophilic groups swelling more than hydrophobic groups. Hydrophobic groups undergo conformational collapse in the presence of water, resulting in reduced swelling capacity of the hydrogel.^59^

In this work, PVA and Gantrez were chosen as the polymeric backbones for hydrogel systems. PVA is an inert polymer featuring repeating polar hydroxyl groups and is therefore a suitable candidate for cross-linking.^60^ Citric acid is nontoxic, and when heated at 130 °C, an esterification reaction occurs between the carboxyl groups in citric acid and the hydroxyl groups in PVA, resulting in a cross-linked network.^34^ PVP was incorporated along with PVA, facilitating the formation of hydrogen bonds between PVA hydroxyl groups and PVP carbonyl groups. Because of the presence of the ring structure in PVP, the structural rigidity of the HFMN is enhanced.^61^ PVA/PVP hydrogels exhibited the lowest swelling capacity that may be attributed to the restricted mobility of polymer chains within the extensively cross-linked network.

The formation of both “normal swelling” and “super swelling” Gantrez hydrogels entailed cross-linking the hydroxyl groups of PEG 10,000 with the free carboxylic acid groups in Gantrez through an esterification reaction at 80 °C for 24 h. The “super swelling” films displayed the largest swelling profile, attributed to the presence of sodium carbonate. This compound led to the formation of sodium salts on free carboxylic acid groups, consequently decreasing the degree of esterification. This served to increase the pore size between cross-links, enabling a greater fluid uptake capacity and resulting in increased swelling.^36^

#### 3.3.2 Permeation of PRA through Swollen Hydrogel Films

**Figure 9:**
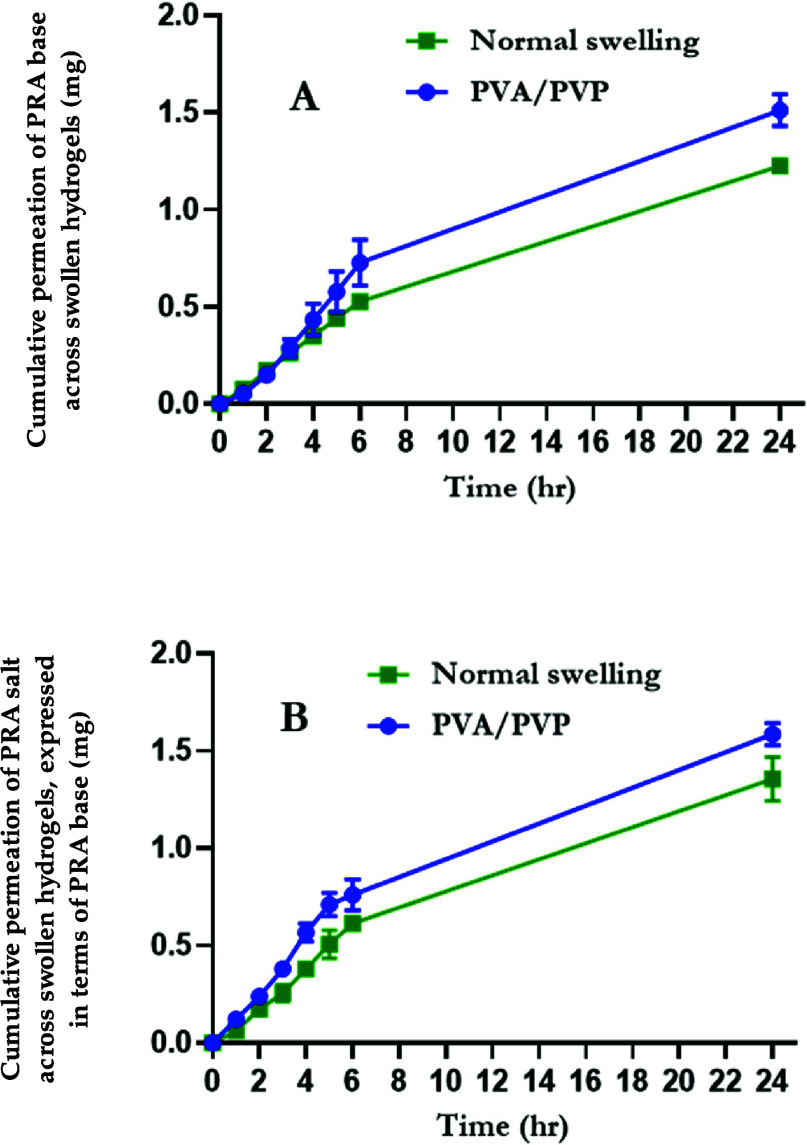
Cumulative permeation of (A) PRA base and (B) PRA salt across swollen PVA/PVP and “normal swelling” Gantrez hydrogel films over 24 h (means ± S.D., *n* = 3).

The permeation of PRA across swollen hydrogel film formulations was assessed using side-by-side diffusion cells, with results presented in Figure 9. The results from the “super swelling” Gantrez formulation are not presented here, as it was observed that, in some cases, the swollen hydrogel film had broken during the side-by-side permeation study. Consequently, the PRA-containing solution had unrestricted passage from the donor compartment to the receptor compartment, with no hydrogel barrier to move through. When considering PRAB, after 24 h, 1.51 ± 0.08 mg of PRA permeated across the PVA/PVP hydrogel into the donor compartment, representing 50.39 ± 2.75% of the initial PRA amount within the donor half-cell of the apparatus. This was significantly higher (*p* = 0.0043) than the cumulative amount that permeated across the “normal swelling” Gantrez film, which was calculated to be 1.23 ± 0.02 mg, equating to 40.86 ± 0.68% of the initial PRA quantity. A similar trend was observed with PRAS, where 1.58 ± 0.06 mg (52.84 ± 1.89%) and 1.36 ± 0.11 mg (45.19 ± 3.71%) permeated through the PVA/PVP and “normal swelling” formulations, respectively, with values expressed in terms of PRAB. Again, significantly more PRA permeated through the PVA/PVP hydrogel (*p* = 0.0336).

It is evident that PRA, being a small molecule, effectively permeates through the pores of both PVA/PVP and “normal swelling” Gantrez-based hydrogel films. Furthermore, the hydrophilic nature of PRA facilitates diffusion through the aqueous hydrogel environment. From the results displayed in Section 3.3.1, it was evident that the “normal swelling” Gantrez hydrogel films exhibited almost 4 times the degree of swelling compared to the PVA/PVP hydrogel films. However, less PRA permeation was observed through the “normal swelling” Gantrez hydrogels. The decreased PRA permeation across the “normal swelling” Gantrez film can possibly be attributed to a chemical interaction. Gantrez forms a negatively charged hydrogel system containing a substantial number of carboxylic acid groups. After cross-linking with PEG, many of these negatively charged carboxylic acid groups remain unreacted.^62^ PRA is a weakly basic drug containing amino groups within its structure. Under low pH conditions, the amino groups become ionized, facilitating interactions with the negatively charged acid groups in Gantrez. The experiment was carried out in PBS, so pH was controlled. However, within the hydrogel, Gantrez will present an acidic environment due to the significant presence of unreacted acid groups. These interactions may potentially hinder the diffusion of PRA through the swollen hydrogel matrix, leading to entrapment of the drug and potentially decreasing delivery to the receptor cell.^34^

#### 3.3.3 Characterization of Hydrogel-Forming Microneedles

**Figure 10:**
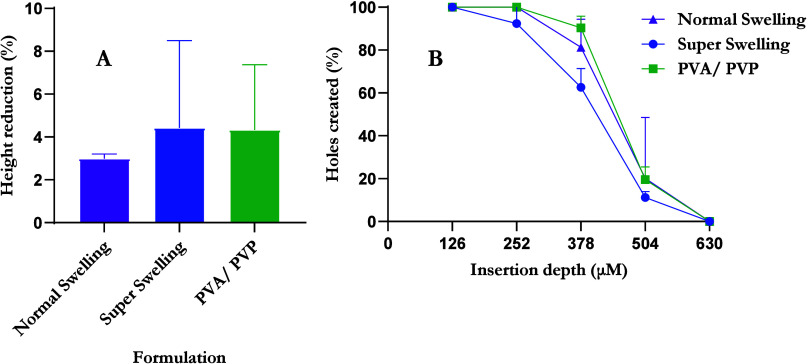
HFMN characterization tests including (A) percentage height reduction (means + S.D., *n* = 5) and (B) percentage insertion profile of needles after insertion into Parafilm M (means + S.D., *n* = 5).

After removal from molds, all HFMN patches, regardless of their formulation, contained 121 conical needles, arranged in an 11 × 11 pattern with a height of approximately 600 μm and interspacing and base width of 300 μm. To ensure that HFMNs could penetrate the stratum corneum, they were placed on Parafilm M that had been folded into eight layers, and a 32 N force was applied for 30 s using the Texture Analyzer. The needle heights before and after insertion were measured, enabling calculation of percentage height reduction, displayed in Figure 10A. The percentage insertion and estimated insertion depth of MNs into Parafilm M were also determined, as shown in Figure 10B.

All three HFMN formulations demonstrated 100% insertion of needles into the first layer of Parafilm M. High levels of insertion were also observed in layer 2 as 100% insertion was achieved for “normal swelling” and PVA/PVP HFMNs, with the “super swelling” formulation exhibiting 92.33 ± 7.88% insertion, representing an insertion depth of approximately 252 μm. The fourth layer insertion percentages were observed to be 20.13 ± 28.40 (“normal swelling”), 11.20 ± 2.76 (“super swelling”), and 19.57 ± 5.88 (PVA/PVP), corresponding to an approximate insertion depth of 504 μm. None of the formulations penetrated the fifth layer of Parafilm M, as its depth of 630 μm exceeded the height of the MNs. The percentage height reductions of the “normal swelling”, “super swelling”, and PVA/PVP formulations were calculated to be 2.99 ± 0.22, 4.42 ± 4.08, and 4.33 ± 3.04%, respectively. Overall, all formulations successfully inserted into the fourth layer of Parafilm M, and less than 10% height reduction was observed, indicating the mechanical properties of the needles.

**Figure 11:**
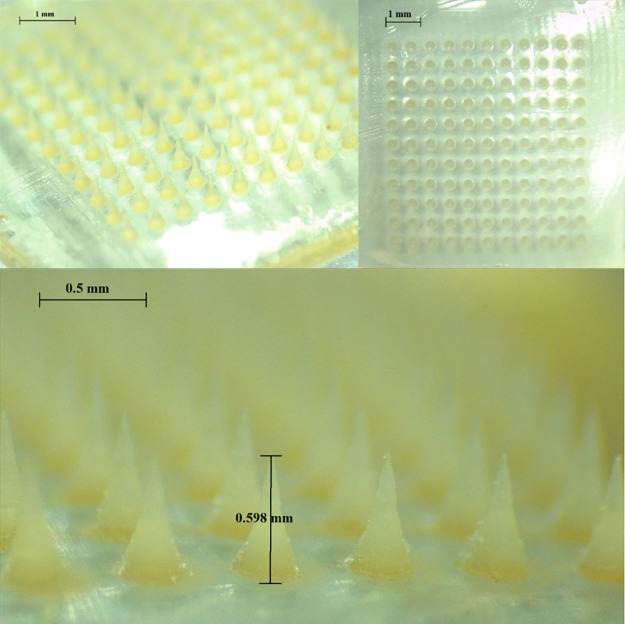
Digital light microscope images of PVA/PVP HFMNs with patches containing an 11 × 11 arrangement of needles (600 μm needle height, conical shape).

PVA/PVP HFMNs, displayed in Figure 11, were chosen for further studies because of their reduced swelling abilities in comparison to the Gantrez formulations, which offer the potential of prolonged drug delivery. Additionally, the PVA/PVP hydrogel was deemed more compatible with PRA as the amino groups in PRA interact with the negatively charged acid groups in Gantrez, potentially reducing the diffusion of PRA through the hydrogel.

#### 3.3.4 Physical Characterization of PRA Base-Containing Directly Compressed Tablets

**Figure 12:**
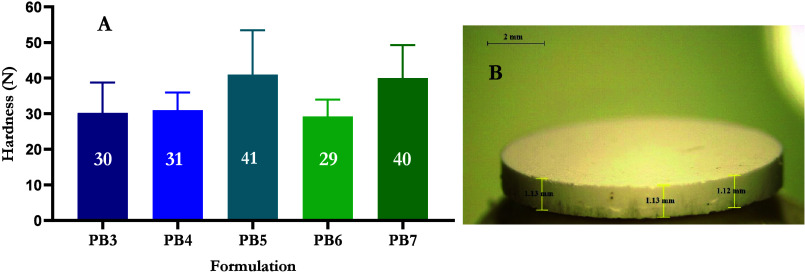
(A) Hardness of PRA base DCTs, with values rounded to the nearest newton (means + S.D., *n* = 10) and (B) a digital light microscope image of DCT formulation PB7.

PRAB-containing DCTs were characterized in terms of visual inspection, diameter, thickness, mass, and hardness, as shown in Table 7 and Figure 12. The objective of assessing the hardness of DCTs was to determine the force necessary to fracture the DCT. This evaluation served as a practical measure of the robustness of each DCT in anticipation of subsequent experiments. Formulations PB1 and PB2 were not included in characterization studies due to their 100% drug content, which hindered proper tablet formation owing to their low mass and absence of necessary excipients. The remaining formulations investigated were prepared using pharmaceutically approved and commonly used excipients for oral tablet formulations.^26,63^

**Table 7:** Physical Characterization of PRAB-Containing DCTs (Mean ± S.D., *n* = 10)

**formulation code**	**diameter (mm)**	**thickness (mm)**	**theoretical mass of DCT (mg)**	**actual mass of DCT (mg)**	**within 7.5% of mean mass?**
**PB3**	9.74 ± 0.27	1.11 ± 0.10	100	99.72 ± 1.00	yes
**PB4**	9.94 ± 0.16	1.08 ± 0.04	100	99.21 ± 1.15	yes
**PB5**	10.31 ± 0.03	1.43 ± 0.02	100	100.13 ± 0.88	yes
**PB6**	9.49 ± 0.46	1.11 ± 0.09	100	99.30 ± 1.39	yes
**PB7**	9.89 ± 0.05	0.99 ± 0.06	100	99.93 ± 0.88	yes

All DCTs containing PRAB exhibited uniform characteristics, with mean masses ranging from 99.21 to 100.13 mg. Prior research has determined that the typical force applied through manual thumb pressure for inserting an MN patch is approximately 32 N.^31^ When contemplating the design of a final HFMN-DCT device, the aspiration is for a one-step application process, whereby both the DCT and HFMN are enclosed within a single package. It has been established that HFMNs, in their dry state, are capable of withstanding a 32 N force without breaking; therefore, it is imperative to ensure that the DCT could likewise endure this force without fracturing. Tablet hardness was used to investigate this. When considering PRAB-containing DCTs, tablet hardness ranged from 29.20 ± 4.76 N for PB6 to 41.00 ± 12.45 N for PB5. Formulation PB6 contained 20% w/w PRA and 80% w/w sorbitol, whereas PB5 contained 20% w/w PRA and 80% w/w crospovidone. The sorbitol used in this study was not spray dried, and it has been recognized that spray dried forms of sorbitol offer greater compression than standard grades, thereby resulting in the formation of harder tablets.^64^ PB6 was modified by reducing sorbitol content to 50% w/w, and MCC was added at a concentration of 30% w/w to form PB7. These alterations significantly enhanced the hardness of the DCT to 40.00 ± 9.25 N (*p* = 0.0488). This may be attributed to the characteristics of MCC as it plays an important role in the bonding strength between adjacent particles, contributing to the mechanical strength of tablets.^65^ Overall, the PRA containing DCTs that showed the most promise were those with hardness values ≥40 N, namely, PB5 and PB7. PB3, PB4, and PB6 were not chosen for ex vivo permeation studies as their hardness was not above 32 N; however, dissolution time and PRA recovery were still investigated for these formulations.

#### 3.3.5 Dissolution Studies and PRA Recovery from DCTs

The dissolution/disintegration time of reservoirs was determined to assess how long it would take for reservoirs to dissolve/disintegrate upon exposure to an aqueous solution. This parameter was indicative of the time frame required for each formulation to dissolve after making contact with interstitial fluid while positioned above a swollen HFMN. In this case, the optimal reservoir would slowly dissolve, facilitating sustained delivery of PRA as it diffuses through the swollen hydrogel matrix. The dissolution times and percentage PRA recovery are displayed in Table 8.

**Table 8:** Dissolution Time and Recovery of PRA from PRAB-Containing DCTs (Means ± S.D., *n* = 4)

**formulation code**	**dissolution time (s)**	**theoretical PRA content (mg)**	**PRA recovery (%)**
**PB3**	83.25 ± 5.44	20	93.17 ± 3.55
**PB4**	216.00 ± 11.93	20	95.05 ± 30.49
**PB5**	9.25 ± 0.96	20	94.87 ± 1.23
**PB6**	171.75 ± 8.88	20	93.23 ± 3.62
**PB7**	182.75 ± 11.09	20	95.24 ± 4.38

When evaluating DCTs, the shortest disintegration time was 9.25 ± 0.96 s, which was observed in the case of DCT PB5, containing the super disintegrant crospovidone, as anticipated. On the other hand, the longest dissolution/disintegration time was 216.00 ± 11.93 s (PB4). However, it is worth noting that the hardness of PB4 was 31 N, slightly below the average thumb pressure of 32 N, potentially leading to fracturing upon application to skin. Consequently, PB7 emerged as a more favorable PRAB-containing choice for further studies, with a disintegration time of 182.75 ± 11.09 s and a hardness of 40 N. The recovery percentages from all DCTs investigated exceeded 93% of their theoretical content. This outcome demonstrates the stability of the reservoirs and implies the lack of any compatibility concerns between PRA and the excipients used.

#### 3.3.6 Ex Vivo Permeation of PRAB from HFMN-DCT Devices

**Figure 13:**
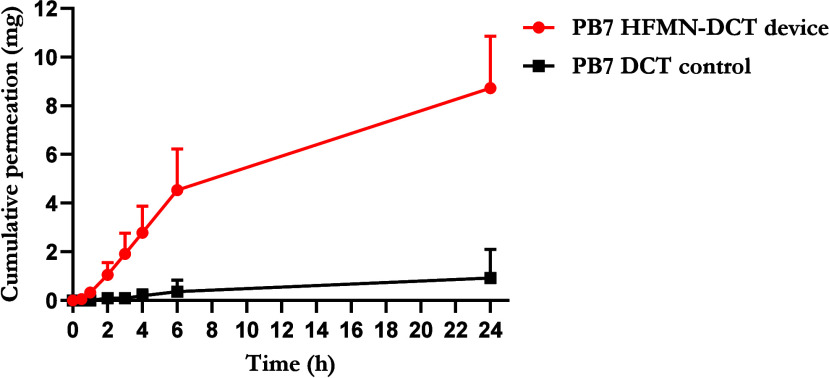
Ex vivo permeation profile of PRAB from PVA/PVP HFMN-DCT devices and their corresponding controls through dermatomed neonatal porcine skin (means + S.D., *n* = 5).

The ex vivo permeation of PRAB from HFMNs in combination with DCTs was investigated. PVA/PVP HFMNs were chosen in this study as they swelled the least compared to the “normal” swelling and “super” swelling Gantrez formulations. This was preferred as it was hoped that the delivery of PRA would be prolonged as a result of less hydrogel swelling. As HFMNs remain intact and swollen upon the uptake of fluid, drug can continuously be delivered through the swollen matrix and across the skin. The permeation of PRAB from the HFMN-DCT device was compared to its corresponding control that was a PRAB-containing DCT (PB7) without the HFMN, and results are displayed in Figure 13. After 24 h, the cumulative permeation of PRA in the receiver compartment was determined to be 8.73 ± 2.13 mg from the PB7 HFMN-DCT device, equating to a delivery efficiency of 45.81 ± 11.116%. This was significantly higher than the control that was 0.93 ± 1.17 mg, representing 4.88 ± 6.15% (*p* = 0.0035). The hygroscopic nature of sorbitol allows it to absorb water, leading to the formation of a wetting front within the tablet. This front penetrates the tablet matrix, causing swelling and weakening of interparticle bonds, ultimately leading to tablet disintegration. MCC, on the other hand, possesses both swelling and capillary action properties, promoting tablet disintegration. The capillary action of hygroscopic MCC involves the absorption of water, which is then transported into the tablet matrix through fine capillary channels.^66^ This capillary action enhances the penetration of water into the tablet, promoting swelling and fragmentation. Additionally, the inherent swelling properties of MCC contributes to the exertion of outward pressure from within the tablet, forming a porous structure within the tablet and further facilitating the disintegration process.^67^ The combination of sorbitol and MCC in PB7 promoted both wicking and swelling mechanisms, ensuring the release of PRA. Unfortunately, because of skin decomposition, it was necessary to terminate the Franz cell experiment after 24 h. However, a prolonged experiment with a duration beyond 24 h could potentially have facilitated the delivery of a greater quantity of PRA from the HFMN-DCT devices. As illustrated in Figure 13, it is evident that the permeation of PRA from the PB7 HFMN-DCT device had not yet reached a plateau, indicating a promising potential for further drug delivery beyond a 24 h time frame.

It is important to mention that the total PRA permeated was superior to previously described conventional patches and MN approaches for the delivery of this drug.^53−55^ As mentioned earlier, one of the reasons is the higher drug loading achieved in the present system.

### 3.4 In Vivo Delivery of Pramipexole

At the beginning of the in vivo study, MN patches were applied to the back of rats in cohorts 2 and 3 without breaking or fracturing in any way, and they were affixed in place employing a backing layer comprising Microfoam surgical tape and Tegaderm. Subsequently, a layer of kinesiology tape was wrapped around the back and abdomen to ensure the secure positioning of both the MN patches and backing layer. Precise application of the kinesiology tape was paramount, as it was essential to ensure that the movement and breathing of each rat were not negatively affected.

Cohort 2 consisted of a total of eight rats, with each rat receiving one PRAS containing DMN. Similarly, cohort 3 comprised eight rats; however, two PRAB containing HFMN-DCT devices were applied to each rat. After 24 h, MN patches were removed from four rats in each cohort, whereas the remaining four rats retained their patches throughout the 5 day study period. The remaining patches were removed after study completion. Gaseous anesthesia was administered to the rats prior to patch removal to prevent discomfort or pain. Upon removal of MN patches after 24 h, the skin on the back of each rat was examined. This examination served three purposes: first, to check for indications of skin reactions; second, to assess the appearance of visible pores resulting from MN insertion; and finally, to ascertain the presence or absence of any undissolved PRA-containing DMNs or DCTs.

**Figure 14:**
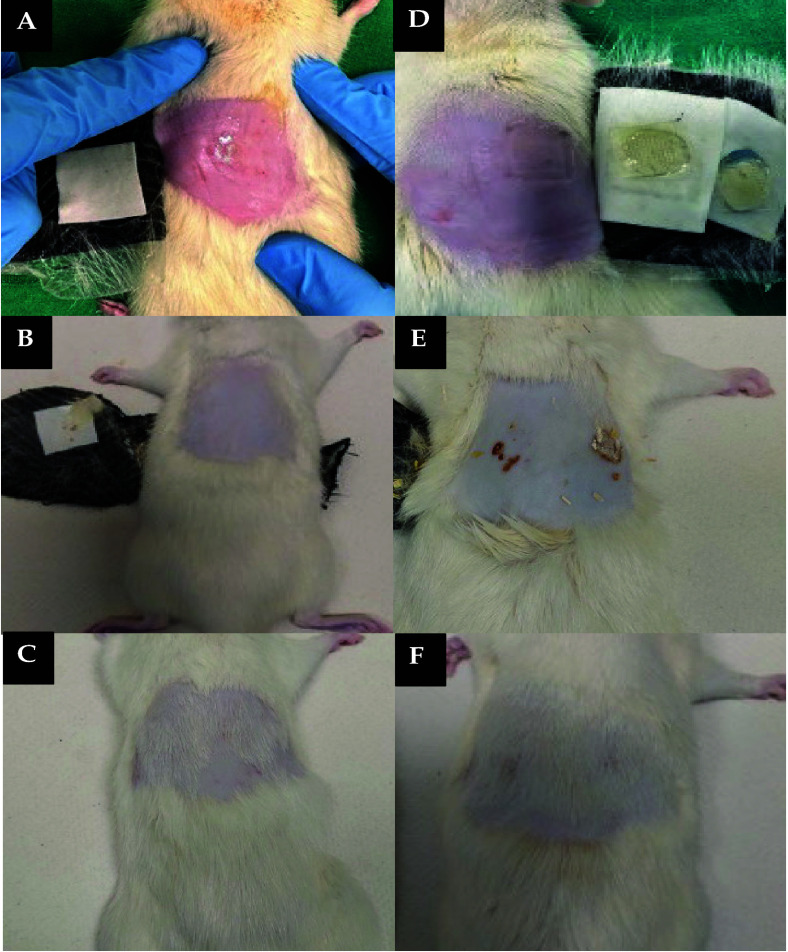
Representative images of application sites of (A) DMNs after 24 h patch removal, (B) DMNs after 5 day patch removal, (C) day 5 skin on rats after DMNs were removed after 24 h, (D) HFMNs after 24 h patch removal, (E) HFMNs after 5 day patch removal, and (F) day 5 skin on rats after HFMNs were removed after 24 h.

Following removal of DMNs at 24 h (Figure 14A), the rats’ skin displayed no visible signs of irritation. Although some MN insertion holes were evident, their visibility was minimal, possibly because of the fast dissolution of DMNs promptly followed by skin recovery. Furthermore, the DMNs appeared to have completely dissolved within 24 h. Nonetheless, the skin’s surface exhibited noticeable moisture, potentially attributed to residual dissolved baseplate. Upon removal of DMNs after 5 days (Figure 14B), again there were no signs of irritation, no remaining DMNs, and no visible pores, indicating the complete dissolution of DMNs and the full restoration of the skin. The skin of the rats from which DMNs were removed after 24 h was continuously observed throughout the study duration. As depicted in Figure 14C, it is apparent that the skin had returned to its normal state, with hair regrowth occurring over the previously shaved area.

HFMNs that were removed after 24 h (Figure 14D) formed prominent pores in the skin as they began to swell and remained intact after insertion. There was minimal skin irritation observed at the site of the adhesive dressing. It was clear that the DCT had not fully dissolved after 24 h, which was an encouraging result as it was hoped that the reservoir would slowly dissolve over 5 days, prolonging drug delivery. Following 5 days of the study, the remaining HFMNs were removed, and again, mild irritation was observed as a result of the adhesive dressing (Figure 14E). This was to be expected given that in a previous investigation that utilized PVA/PVP HFMNs for the delivery of the cytotoxic drug methotrexate, there were no indications of redness or swelling at the site of MN application even after a 24 h period following HFMN removal. It is noteworthy that in this earlier study, the HFMN patches were affixed for a duration of 48 h.^34^ In prior research involving human volunteers, it was observed that the skin barrier function recovered within 24 h following the removal of “normal swelling” Gantrez HFMNs when they had been in the skin for 6 h and had significantly swelled in volume. After patch removal, only minor erythema was occasionally noted, a condition that consistently resolved within 48 h, with no subsequent adverse events reported during follow-up.^35^ In a separate study again employing “normal swelling” Gantrez HFMNs, some participants exhibited erythema immediately after HFMN removal; however, this effect completely dissipated within 1 h.^51^ To the best of available knowledge, there are no published studies documenting skin observations following the application of HFMNs for 5 days. However, the safety profile of HFMNs has been extensively documented,^24,68,69^ and the specific ingredients, PVA, PVP, and citric acid, have long histories of safe use.^70−72^

Both MCC and sorbitol have proven to be safe on the skin; therefore, the reaction may be due to the quantity of PRA loaded into the DCT.^73,74^ Once again, the skin of the rats subjected to HFMN removal after 24 h underwent continuous monitoring over the entire course of the study. As illustrated in Figure 14F, it is evident that no signs of infection were present and the area that had been previously shaved exhibited hair regrowth. It was particularly encouraging that even though MNs punctured the protective barrier provided by the stratum corneum and no prior cleansing of the skin was performed before MN application, there were no visible signs of infection. This could be ascribed to the repair and defensive mechanisms of the skin.^69^

**Figure 15:**
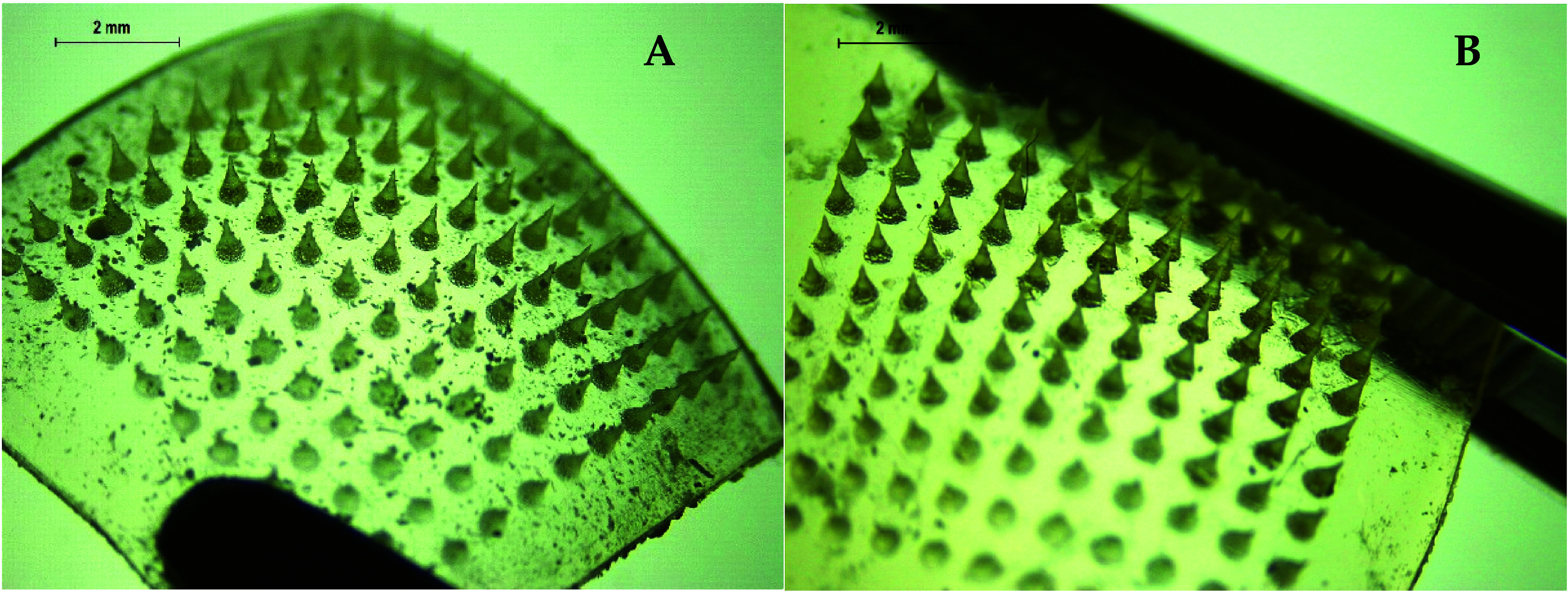
Representative images of HFMNs removed intact after application times of (A) 24 h and (B) 5 days.

HFMNs were removed intact after 24 h and 5 day application times as displayed in Figure 15A,B. The successful removal of intact needles provides reassuring evidence that polymer deposition in skin had not occurred. The removed HFMNs were cut into smaller fragments and immersed in a solution comprising water and ACN, with the aim of quantifying PRA within the hydrogel matrix. The levels of PRA were not detectable using the validated RP-HPLC-UV method, as no peak was observed. This may be attributed to entrapment of PRA within the hydrogel proving difficult to remove, or very little PRA may have been retained because of its hydrophilic nature. The residual DCTs that accompanied the removed HFMNs were also quantified at 24 h and 5 day time points. Before the study commenced, each DCT prepared contained approximately 19.05 mg of PRAB. When HFMN-DCT devices were removed after 24 h and 5 days of application, there was a significant difference in the quantity of PRA remaining in DCTs, with 9.02 ± 0.83 and 1.79 ± 0.81 mg PRA quantified, respectively (*p* < 0.0001). This outcome holds promise, indicating that PRA delivery may have extended beyond the initial 24 h and potentially continues for 5 days.

It must be acknowledged that retrieving the entirety of the remaining PRA proved challenging, as small amounts of the DCT adhered to the backing layer and kinesiology tape. Regarding DMNs, quantifying the remaining PRA proved impossible, as the patch appeared to be completely dissolved after 24 h, leaving behind a sticky residue. This residue was not present at the 5 day patch removal time point. It was difficult to collect the residue, and it is possible that a portion of it might have been absorbed by the backing layer.

#### 3.4.1 Pharmacokinetic Analysis

Pharmacokinetic parameters for each cohort, such as *C*_max_ and *T*_max_, were determined by carefully examining the raw data. Furthermore, the total PRA exposure (AUC_0–120_) was calculated. Given that the expected amount of PRA delivered was comparable in each cohort of rats, both MN cohorts were separately compared to the oral control group.

**Figure 16:**
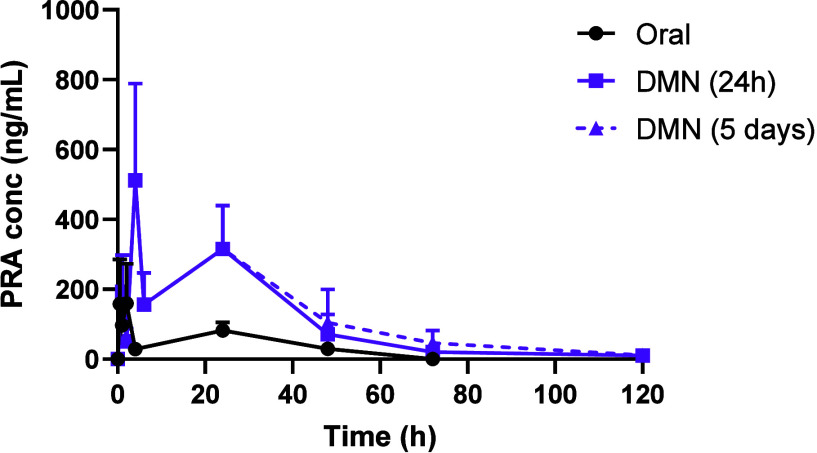
PRA plasma profile of rats from the oral and DMN cohorts over a 5 day in vivo study (oral cohort: means + S.D., *n* = 6 for 0–24 h and *n* = 3 for 24–120 h, DMN cohort: means + S.D., *n* = 8 for 0–24 h and *n* = 4 from 24 to 120 h).

The in vivo plasma profile of PRA from rats in cohort 1 (oral) and cohort 2 (DMNs) is displayed in Figure 16. For orally administered PRA, the maximum plasma concentration of 159.32 ± 113.43 ng/mL was detected 2 h after administration, with *T*_max_ of 2 h in line with previous studies.^75^ There was no significant difference in the *C*_max_ value observed for plasma concentrations in DMNs with the highest plasma concentration of 511.00 ± 277.24 ng/mL obtained at 4 h (*p* = 0.0970). The delayed *C*_max_ value for DMNs compared to the oral group may be attributed to the time taken for dissolution of the DMN followed by drug dissolution and absorption by the dermal microcirculation. In terms of the overall PRA exposure, the AUC_0–120 h_ obtained following oral administration was 3219 ± 518 ng/mL·h. Within the DMN cohort, rats were divided into two groups: one in which patches were removed from four rats 24 h after application and the other in which patches on the remaining four rats were kept in place for 5 days. For both groups within the cohort, blood samples were taken throughout the 5 day period. Consequently, the AUC_0–120 h_ for rats with patches retained for 24 h was 12167 ± 2337 ng/mL·h, and this value was not significantly different to rats that kept their DMN patches on for 5 days, which yielded an AUC_0–120 h_ of 13930 ± 2841 ng/mL·h (*p* = 0.4768). At the 24 h time point, all the delivered PRA from the DMN patches may have been deposited in skin. This was evidenced by the MRT_0-inf_obs_ for PRA in which no significant difference was observed between both DMN cohorts (*p* > 0.05).Therefore, there was no additional benefit to leaving the DMN patches on for 5 days. However, DMN patches that were kept on for 24 h and 5 days both achieved significantly higher AUC_0-inf_obs_ values compared to the oral group, with *p* < 0.05 in both cases. This suggests that a gel reservoir may have formed in the skin after application of DMN patches, leading to the somewhat sustained plasma profiles observed.

**Figure 17:**
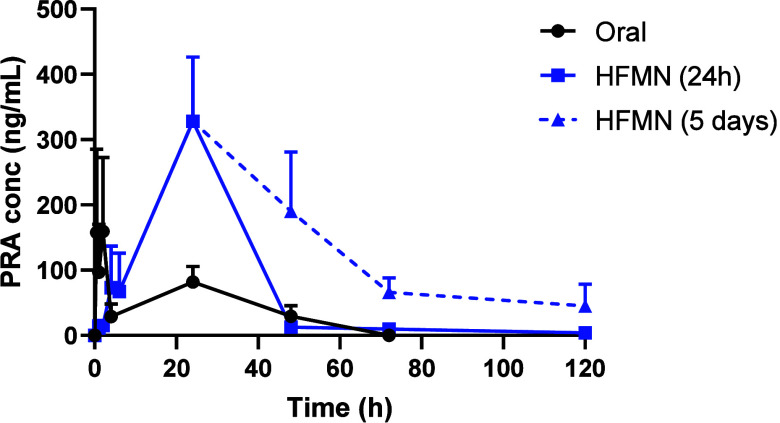
PRA plasma profile of rats from the oral and HFMN cohorts over a 5 day in vivo study (oral cohort: means + S.D., *n* = 6 for 0–24 h and *n* = 3 for 24–120 h, HFMN cohort: means + S.D., *n* = 8 for 0–24 h and *n* = 4 from 24 to 120 h).

Figure 17 showcases the in vivo plasma profiles of PRA for rats in cohort 1 (oral) and cohort 3 (HFMNs). Again, for orally administered PRA, *T*_max_ was observed at 2 h with a peak plasma concentration of 159.32 ± 113.43 ng/mL and an AUC_0–120 h_ of 3219 ± 518 ng/mL·h. HFMN-DCT devices achieved a *C*_max_ value of 328.30 ± 98.04 ng/mL at 24 h. Although the peak plasma concentrations of the oral and HFMN groups were not significantly different (*p* = 0.0877), they were observed at noticeably different times. In cohort 3, the peak plasma concentration of PRA was detected at 24 h. This delay in *C*_max_ may be attributed to the time required for the HFMN to imbibe aqueous fluid, swell, and subsequently dissolve the DCT reservoir positioned on top. Following this, PRA gradually diffused through the swollen hydrogel matrix, ultimately leading to PRA absorption into the dermal microcirculation. Regarding AUC_0–120 h_, values of 8502 ± 1578 and 15767 ± 2414 ng/mL·h were obtained for rats with patches removed after 24 h and 5 days, respectively. This represents a significant difference (*p* = 0.02), as the AUC almost doubled when patches remained in place for 5 days. This is a very encouraging result and aligns with the reduced PRA content in DCTs removed at 5 days compared to those removed after 24 h. This emphasizes the effectiveness of choosing a slowly dissolving DCT combined with a PVA/PVP based HFMN for extending PRA delivery over a 5 day period. Furthermore, in terms of AUC_0-inf_obs_ values, both groups within the HFMN cohort significantly outperformed the oral control cohort, with *p* < 0.05 in both cases, highlighting that even patches retained for 24 h provided greater PRA exposure.

Details of the pharmacokinetic parameters from all cohorts are summarized in Table 9. *C*_max_ for the oral group was detected at 2 h. Although not statistically significant, the oral *C*_max_ value was lower than the other two cohorts and was possibly affected by food administered. Whereas PRA absorption in humans is not affected by food, a decrease in *C*_max_ has been observed in rats in the presence of food.^76,77^ For the DMN cohort, *C*_max_ was observed at 4 h. The PRA plasma levels then decreased and increased again at 24 h. The initial peak observed at 4 h may have been due to the absorption of PRA in the needle tips and some of the MN baseplate. The second peak at 24 h may have been attributed to the absorption of remaining PRA that was present in the baseplate of the DMN. Because the baseplate layer lies above the skin, it takes longer to dissolve, resulting in slower absorption of PRA into the bloodstream. During the initial 6 h of the study, the plasma levels of PRA in rats that received HFMNs remained consistently low and began to rise after 6 h, reaching a peak at 24 h. Notably, when HFMN patches were removed after 24 h, PRA plasma levels declined rapidly in comparison to the gradual decrease observed when patches were in place for 5 days. This was also evident in the MRT_0-inf_obs_ values, which were significantly greater for HFMNs left in place for 5 days (*p* < 0.05). However, it is worth emphasizing that in both the DMN and HFMN cohorts where patches were removed after 24 h, PRA was still detected in plasma even at 120 h, with concentrations of 10.12 ± 4.59 and 3.88 ± 1.05 ng/mL, respectively. This suggests the possible formation of an intradermal drug depot enabling the absorption of PRA after patch removal, which is an interesting observation given the hydrophilic nature of both the salt and base forms of PRA. In terms of the HFMN-DCT device, any depot formed post removal at 24 h could possibly be due to the slightly lower water solubility of the base, potentially leading to some coming out of solution and then redissolving over time. When comparing the patches that were kept in place for 5 days, PRA concentrations of 11.61 ± 19.14 and 45.10 ± 33.25 ng/mL were found for DMNs and HFMNs, respectively. There was no significant difference in the concentrations found at 120 h for DMN patches removed after 24 and 120 h (*p* = 0.8851). However, significantly more PRA was detected at 120 h for HFMNs that were kept in place for 5 days vs the HFMNs taken off after 24 h (*p* = 0.048), emphasizing that prolonged PRA delivery was achieved.

**Table 9:** In Vivo Plasma Pharmacokinetic Parameters of PRA after Oral, DMN, and HFMN Administration to Sprague–Dawley Rats^a^

**parameter**	**unit**	**cohort 1 oral control**	**cohort 2 DMN** (24 h)	**cohort 2 DMN** (5 days)	**cohort 3 HFMN** (24 h)	**cohort 3 HFMN** (5 days)
*t*_max_	h	2	4	4	24	24
*C*_max_	ng/mL	159.32 ± 113.43	511.00 ± 277.24	511.00 ± 277.24	328.30 ± 98.04	328.30 ± 98.04
MRT_0–inf_obs_	h	29.85 ± 12.83	33.70 ± 2.68	31.98 ± 3.34	30.75 ± 1.45	50.19 ± 7.94
AUC_0–inf_obs_	ng/mL·h	3686.13 ± 1422.06	13580.64 ± 3604.39	14465.05 ± 2337.14	7901.04 ± 1630.49	17697.64 ± 5380.92
AUC_0–120 h_	ng/mL·h	3,219 ± 518	12,167 ± 2337	13,930 ± 2841	8,502 ± 1578	15,767 ± 2414

a DMN (24 h) relates to DMN patches that remained in place for 24 h. Similarly, DMN (5 days) relates to DMN patches kept in place for 5 days. HFMN cohorts also follow this trend (oral cohort: means + S.D., *n* = 6 for 0–24 h and *n* = 3 for 24–120 h; DMN cohort: means + S.D., *n* = 8 for 0–24 h and *n* = 4 for 24–120 h; and HFMN cohort: means + S.D., *n* = 8 for 0–24 h and *n* = 4 for 24–120 h).

## 4 Conclusions

This work provides crucial proof-of-concept evidence that transdermal delivery using MN technology can sustain the delivery of the hydrophilic drug PRA. Furthermore, the work presented here highlights the versatility of polymeric MN patches as PRA can be incorporated into DMNs, producing mechanically strong needles capable of bypassing the outermost layer of the skin. Polymeric HFMNs can also be used, where the composition and cross-linking density can be altered according to the desired outcome. HFMNs in combination with PRAB-containing DCTs have proven to be a suitable device for sustained PRA delivery. Following an in vivo study conducted on rats, both MN cohorts achieved sustained plasma levels of PRA over 5 days. In contrast, the last detection of orally administered PRA in plasma occurred at 48 h. Following the in vivo study, the HFMN that remained in place for 5 days demonstrated the most promising performance among all investigated formulations. This formulation exhibited the highest PRA plasma concentration after 5 days of application and provided significantly greater PRA exposure compared to all other cohorts evaluated. Furthermore, the swollen HFMN was removed intact after the 5 day period. It is acknowledged that the development of a suitable applicator would be beneficial for patients to ensure the reproducibility of patch application, especially for those with more advanced Parkinson’s disease. Additionally, evaluating the storage stability of the formulations is a critical step that should be addressed during future research. Overall, the work detailed here highlights the potential of polymeric MNs to deliver potent hydrophilic compounds resulting in sustained levels of drug in plasma, potentially enhancing patient compliance and quality of life by decreasing dosing frequency.

## Data Availability

Data will be made available on request.

## Abbreviations

PRA: pramipexole
DMN: dissolving microneedle
PVA: poly(vinyl alcohol)
PVP: poly(vinylpyrrolidone)
HFMN: hydrogel-forming microneedle
DCT: directly compressed tablet
PD: Parkinson’s disease
HPLC: high-performance liquid chromatography
